# Field‐Effect Enhancement of Non‐Faradaic Processes at Interfaces Governs Electrocatalytic Water Splitting Activity

**DOI:** 10.1002/advs.202403206

**Published:** 2024-06-27

**Authors:** Ning Wen, Haihua Wang, Qilu Liu, Kepeng Song, Xiuling Jiao, Yuguo Xia, Dairong Chen

**Affiliations:** ^1^ National Engineering Research Center for Colloidal Materials School of Chemistry and Chemical Engineering Shandong University Jinan Shandong 250100 P. R. China; ^2^ State Key Laboratory of Crystal Materials Shandong University Jinan Shandong 250100 P. R. China

**Keywords:** field effect, HER and OER, interfacial adsorption modulation, non‐Faradaic process, water electrocatalysis

## Abstract

Recognizing the essential factor governing interfacial hydrogen/oxygen evolution reactions (HER/OER) is central to electrocatalytic water‐splitting. Traditional strategies aiming at enhancing electrocatalytic activities have mainly focused on manipulating active site valencies or coordination environments. Herein, the role of interfacial adsorption is probed and modulated by the topological construct of the electrocatalyst, a frequently underestimated non‐Faradaic mechanism in the dynamics of electrocatalysis. The engineered Co_0.75_Fe_0.25_P nanorods, anchored with FeO_x_ clusters, manifest a marked amplification of the surface electric field, thus delivering a substantially improved bifunctional electrocatalytic performance. In alkaline water splitting anion exchange membrane (AEM) electrolyzer, the current density of 1.0 A cm^−2^ can be achieved at a cell voltage of only 1.73 V for the FeO_x_@Co_0.75_Fe_0.25_P|| FeO_x_@Co_0.75_Fe_0.25_P pairs for 120 h of continuous operation at 1.0 A cm^−2^. Detailed investigations of electronic structures, combined with valence state and coordination geometry assessments, reveal that the enhancement of catalytic behavior in FeO_x_@Co_0.75_Fe_0.25_P is chiefly attributed to the strengthened adsorptive interactions prompted by the intensified electric field at the surface. The congruent effects observed in FeO_x_‐cluster‐decorated Co_0.75_Fe_0.25_P nanosheets underscore the ubiquity of this effect. The results put forth a compelling proposition for leveraging interfacial charge densification via deliberate cluster supplementation.

## Graphical abstract

The swift depletion of reactants at the electrocatalyst surface accentuates the pivotal role of reactant adsorption at the catalyst/electrolyte boundary, thereby rendering this non‐Faradaic event the rate‐limiting stage, particularly under conditions of elevated current density. In this work, it is unveiled how electrocatalytic water splitting can be finely regulated through the alteration of interfacial electric fields, a consequence of the catalyst's inherent topological configuration.

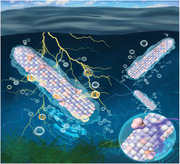

## 1 Introduction

The conversion of renewable energy into clean, storable fuels plays a vital role in meeting global energy demands while reducing the effects on the environment.^[^ ^1^, ^2^ ^]^ Electrocatalytic water splitting is a pivotal technology in this realm, promising substantial potential for sustainable hydrogen production and advancing carbon‐neutral energy strategy.^[^ ^3^, ^4^, ^5^, ^6^, ^7^ ^]^ Nonetheless, the deployment of electrolytic hydrogen production on an industrial scale is delayed by obstacles, including the requirement for catalysts that are simultaneously efficient, stable, cost‐effective, consistent provision of renewable energy, and the development of robust membrane electrolysis cells.^[^ ^8^, ^9^, ^10^ ^]^ Among others, the design and optimization of electrocatalysts directly affect the efficiency and industrialization development of electrolytic hydrogen production, thus holding a paramount position in the industrialization process.^[^ ^11^ ^]^

Currently, catalyst design depends on manipulating binding energies through the strategic alteration of the charge distribution at the active sites, aiming to refine charge transfer and the adsorption/desorption kinetics of intermediates.^[^ ^12^, ^13^, ^14^ ^]^ This involves a series of modulation strategies, ranging from monophasic approaches, including the control of spin, defect, and amorphous states, along with multiphasic approaches such as heterojunction, single‐atom, and metal‐support strong interactions (SMSI).^[^ ^15^, ^16^, ^17^, ^18^, ^19^ ^]^ An example of these strategies is the formation of heterojunctions, which integrate different materials to form new interfacial properties, imparting distinct electronic features at the junctions, such as enhanced interface charge transfer and optimized valence states, ultimately lowering the energy barriers of reactions.^[^ ^20^, ^21^, ^22^ ^]^ However, there are inherent limitations to the charge transfer capabilities of heterojunctions, and the potential active sites at these interfaces may end up being obscured.^[^ ^23^ ^]^ In addition, the complexities and limitations of electron structure refinement reflect the difficulties of devising catalysts that can operate efficiently.^[^ ^24^ ^]^ Specifically, at high current densities, the rapid consumption of reactants at the catalyst's surface results in OH^−^/H_2_O adsorption (a non‐Faradaic process) that becomes the rate‐determining step.^[^ ^25^, ^26^, ^27^ ^]^ Merely manipulating the electronic structure of active sites falls short of significantly enhancing the catalyst's adsorptive properties for reactants to satisfy the demands imposed by high current densities, heralding the need for innovative approaches in electrocatalyst design.

Electrocatalytic water‐splitting operates within a triphasic system comprising solid electrodes, electrolytes, and gas products.^[^ ^28^ ^]^ Insights from the Gouy‐Chapman‐Stern model of the electric double layer (EDL) reveal that ion adsorption at the electrode interface results in a compact layer formation, with a subsequent ion distribution extending into the solution as described by the Boltzmann distribution.^[^ ^29^, ^30^, ^31^ ^]^ This condition forms a gradient of ions orthogonal to the electrode surface. When a negative bias is applied, an enhanced attraction of cations and a repulsion of anions ensue. It is thus imperative to concentrate on augmenting the quantities of OH^−^/H_2_O across both the compact and diffuse layers to improve catalytic efficiency in alkaline electrolytes.^[^ ^32^, ^33^ ^]^ Besides, given the extensive dipole moment characteristic of water molecules, the presence of a more pronounced electric field at the catalyst/electrolyte interface is likely to favor the selective orientation and dense adsorption of H_2_O molecules, corroborating theories derived from the EDL model ^[^ ^34^ ^]^ Therefore, such a focused electric field can become an effective catalyst for water splitting reactions. Furthermore, the inherent predisposition of water to form tenacious hydrogen bonds necessitates a nuanced balance between water‐surface and water‐water interactions on the catalyst/electrolyte interface, where water adsorption energy echoes the strength of these hydrogen bonds. ^[^ ^35^ ^]^ Similarly, the adsorption of OH^−^ ions, especially in alkaline electrolytes, is also at the mercy of surface electric fields. Given that OH^−^ ions exhibit strong hydrogen bonding propensities with water, their efficient adsorption also enhances water co‐adsorption, rendering the electric field a key role in reactant accumulation and optimization of reaction barriers.^[^ ^36^ ^]^ Therefore, based on the above‐discussed electrocatalytic water splitting reactants, H_2_O and OH^−^ in alkaline media have polar and dipole moments, and modulation of the interfacial electric field can optimize the binding energies of the adsorbed reactants to influence the catalytic efficiency. In addition, modulation of the interfacial electric field can promote the escape of H_2_/O_2_ to rapidly expose occupied active sites due to changes in surface polarity. This implies that the interfacial electric field modulation strategy is independent of the conventional water‐splitting adsorption mechanism, the scaling relationship of which has fundamental limitations on the catalyst performance.

In this Article, we used the properties of FeO_x_ clusters anchored on Co_0.75_Fe_0.25_P bimetallic phosphide nanorods to probe the influence of cluster morphology on the distribution of the surface electric field. Particularly, we explored the fine electric fields locally and scrutinized the adsorption dynamics of OH^−^/H_2_O at the FeO_x_@Co_0.75_Fe_0.25_P junctions. Critical electrochemical testing evaluating catalytic activity and structural integrity indicated that the FeO_x_@Co_0.75_Fe_0.25_P demonstrated superior HER and OER bifunctional activity alongside sustained durability under rigorous current density conditions. In situ electrochemical spectroscopy illustrates that the FeO_x_@Co_0.75_Fe_0.25_P surface avidly binds significant volumes of OH^−^/H_2_O, revealing the underlying mechanism of its efficient catalytic ability. Corroborating theoretical calculation, valence states, and coordination structure analysis, we validated that the observed catalytic activity improvements are predominantly due to the reactant adsorption induced by the enhanced surface electric field, which is a direct consequence of the topological manipulation of the electrocatalyst. In conclusion, the interfacial electric field induced by the topological morphology promotes the adsorption of polar H_2_O and OH^−^ and the desorption and escape of H_2_/O_2_ in alkaline media improves the catalytic performance and provides a powerful tool for modulating the electrocatalyst activity. This topological structure‐induced enhancement of the surface electric field transcends the inherent limitations of traditional charge transfer theory, offering a new perspective for regulating material adsorption on the electrocatalyst/electrolyte interface.

## 2 Results and Discussion

### 2.1 Finite Element Simulation for Surface Charges and OH^−^/H_2_O Absorption

**Figure 1:**
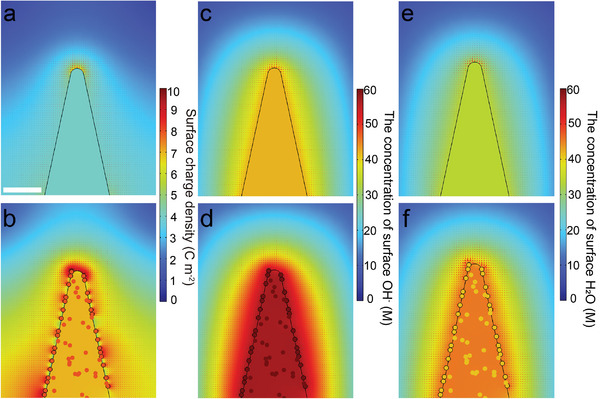
Charge density distribution on the surface of a) Co_0.75_Fe_0.25_P and b) FeO_x_@Co_0.75_Fe_0.25_P. Surface density distribution of OH^−^ on the electrode surface of c) Co_0.75_Fe_0.25_P and d) FeO_x_@Co_0.75_Fe_0.25_P. Surface H_2_O density distribution on the electrode surface of e) Co_0.75_Fe_0.25_P and f) FeO_x_@Co_0.75_Fe_0.25_P. The scale bar represents 50 nm.

Based on COMSOL multiphysics field finite element simulations, the local electric field and the amount of adsorbates on the surface of nanorods were investigated.^[^ ^37^, ^38^ ^]^ Initially, we conducted simulations to investigate the smooth CoP nanorods with varying Fe doping concentrations (Figure S1, Supporting Information). We identified an intriguing trend: an increase in Fe doping in CoP nanorods corresponds to increased surface charge density on the nanorods. Notably, the absence of a significant localized electric field can be attributed to the smooth surface characteristics, lack of curvature, and uniform Co/Fe distribution regardless of the increased charge density. Given these findings, we selected Co_0.75_Fe_0.25_P nanorods, demonstrating the highest surface charge, for further modification with surface clusters. We constructed cluster‐loaded nanorods, simulated their surface charge densities, and identified a significant increase in the local electric field surrounding each cluster, increasing surface charge density to drive the growth in electrocatalytic activity (**Figure** **1**a,b). To elucidate the relationship between the local electric field and overall water‐splitting activity, we quantified the impact of the localized field on OH^−^/H_2_O adsorption, which was achieved using the Gouy‐Chapman‐Stern model, considering adsorbates concentration within the electrode's bilayer Helmholtz layer.^[^ ^39^ ^]^ Notably, the cluster‐enhanced local field led to a ≈2.5‐fold rise in OH^−^ adsorption, promoting the OER process, especially under high current densities and surface polarization voltages (Figure 1c,d; Table S1, Supporting Information). Furthermore, given the crucial role of water adsorption in alkaline HER, we designed a model to determine water molecule adsorption (Figure 1e,f). Unsurprisingly, cluster introduction significantly increased water adsorption, making modified nanorods with enhanced electric fields more efficient in electrocatalysis. Consequently, these cluster‐modified nanorods with enhanced local electric fields demonstrated a more refined “division of labor” in alkaline electrolytes: the clusters more readily adsorbed OH^−^ ions, while the nanorod surface primarily attracted H_2_O molecular, together enhancing OH^−^/H_2_O adsorption kinetics. Based on these simulation results, we posit that the interfacial electric field can be modulated by the topological structure of the electrocatalyst. Particularly, the initial non‐Faraday process of OH^−^/H_2_O absorption could be significantly improved by the local interfacial electric field regulation, especially prominent under conditions of large polarized current density, which may make the non‐Faraday process of OH^−^/H_2_O absorption the rate‐determining steps in electrocatalytic water splitting.

### 2.2 Synthesis and Physicochemical Characterizations

**Figure 2:**
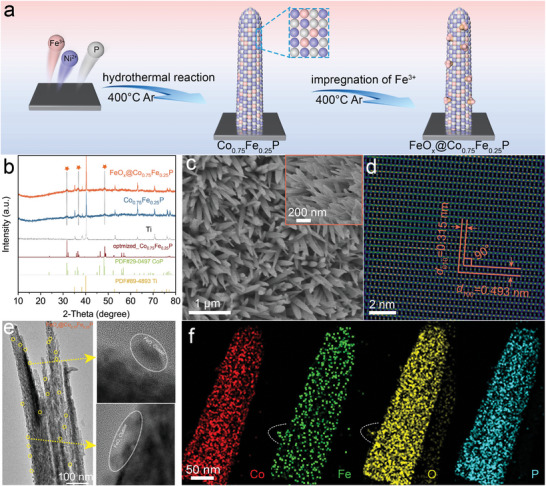
a) Synthesis routes of FeO_x_@Co_0.75_Fe_0.25_P electrodes. b) The powder X‐ray diffraction patterns. c) The SEM images of FeO_x_@Co_0.75_Fe_0.25_P. d) HAADF–STEM image. e) HRTEM image. f) elemental mapping images.

Supplementary experiments were conducted to validate the enhanced interfacial electric field theory modulated by the topological structure as in simulation. We synthesized FeO_x_@Co_0.75_Fe_0.25_P by employing commercially available Ti fiber felt as a support due to its superior conductivity, expansive surface area, and gas transport layer potential for membrane electrode assembly (**Figure** **2**a).^[^ ^40^, ^41^ ^]^ Specifically, the Fe‐doped CoOOH nanorods were grown directly on the Ti fiber felt via a simple hydrothermal method and were later converted to Fe‐doped CoP adopting the inherited Co_1‐x_Fe_x_P morphology by a phosphorization route under an Ar atmosphere at 350 °C. The constructed bimetallic phosphides with varied Fe doping concentrations showed similarities in structure to CoP and FeP, as confirmed by X‐ray diffraction analysis revealing crystalline CoP (JCPDS, no. 29–0497) with no superfluous peaks (Figure S2, Supporting Information). The nanorods were later exposed to a Fe^3+^ solution and calcined to generate FeO_x_ nanocluster‐modified Co_1‐x_Fe_x_P nanorods, denoted as FeO_x_@Co_1‐x_Fe_x_P. The scanning electron microscopy (SEM) images revealed well‐defined nanorod shapes of FeO_x_@Co_1‐x_Fe_x_P and consistency in size for phosphides when the Fe percentage was below 25% (Figure S3, Supporting Information). The polarization‐electric field hysteresis loops (*P*‐*E* loops) confirmed that surface polarization of Co_0.75_Fe_0.25_P emerges as the most impactful in *P*‐*E* loops with varying Fe doping amounts, higher than *P*_r_ and *P*_s_ values compared to CoP and Co_0.875_Fe_0.125_P (Figure S4, Supporting Information). Simultaneously, the surface Zeta potential of CoP exhibits a discernible volcanic trend against Fe doping, reflecting proportional changes noted in HER and OER performance with Fe content alterations (Figure S4, Supporting Information). Such correlation emphasizes the central role of Fe content modulation in water splitting. Considering the effect of substrate topography on interfacial electric field distribution, the study utilized CoP, Co_0.875_Fe_0.125_P, and Co_0.75_Fe_0.25_P nanorods to investigate surface charge density and the resulting adsorption of OH^−^/H_2_O, deploying finite element analysis simulation. We thereby constructed three nanorod‐based models to elucidate the catalytic enhancements afforded by modified surface charge densities (Figure S5, Supporting Information). The finite element analysis simulation further affirmed the surface polarization trend, where more OH^−^/H_2_O were adsorbed on the surface of Co_0.75_Fe_0.25_P nanorods in alkaline electrolyte due to the altered surface charge density. Consequently, Co_0.75_Fe_0.25_P was selected for in‐depth analysis in this study.

Comparative X‐ray diffraction studies between pristine and FeO_x_‐clustered nanorods indicate congruent peak positions, hinting at the preservation of lattice constants across both variants and underscoring the uniformity and diminutive nature of the FeO_x_ clusters (Figure 2b). Besides, the SEM image showcases the densely packed Co_0.75_Fe_0.25_P nanorods anchoring steadfastly to self‐supporting Ti fiber matrices (Figure 2c). The magnified imagery depicts rods measuring ≈ 800 nm in length by 60 nm in diameter. The nanorod arrays on a self‐sustaining electrode framework offer an expansive surface conducive to accelerated mass transport, particularly at heightened current densities. Moreover, the high‐angle annular dark‐field STEM (HAADF‐STEM) maps showed 0.315 and 0.493 nm lattice stripes congruent with the crystallographic surfaces of CoP, confirming dominant (001) facets for Co_0.75_Fe_0.25_P nanorods (Figure 2d). Meanwhile, the high‐resolution electron microscopy (HR‐TEM) image confirmed that FeO_x_ nanoclusters anchored on the surface of Co_0.75_Fe_0.25_P nanorods reveal size of ≈ 4 nm (Figure 2e), and the STEM energy‐dispersive X‐ray mapping also evidences the FeO_x_@Co_0.75_Fe_0.25_P heterostructure (Figure 2f), indicative of the successful construction of the interfacial structure of FeO_x_ clusters and Co_0.75_Fe_0.25_P nanorods. In addition, we added a relevant structural characterization of the FeO_x_ state. XRD and XPS of FeO_x_ individually loaded on Ti carriers are shown in Figure S6 (Supporting Information). The diffraction peaks of XRD correspond precisely to Ti (PDF#89‐4893), suggesting that FeO_x_ clusters are small, dispersed, and amorphous, which helps to provide a large number of catalytically active sites. The binding energy of FeO_x_ is shifted to a higher field compared to FeO_x_@Co_0.75_Fe_0.25_P, suggesting that the higher valence of Fe in the FeOx clusters facilitates OER catalytically active sites.

### 2.3 The Electrocatalytic Water‐Splitting Performances

**Figure 3:**
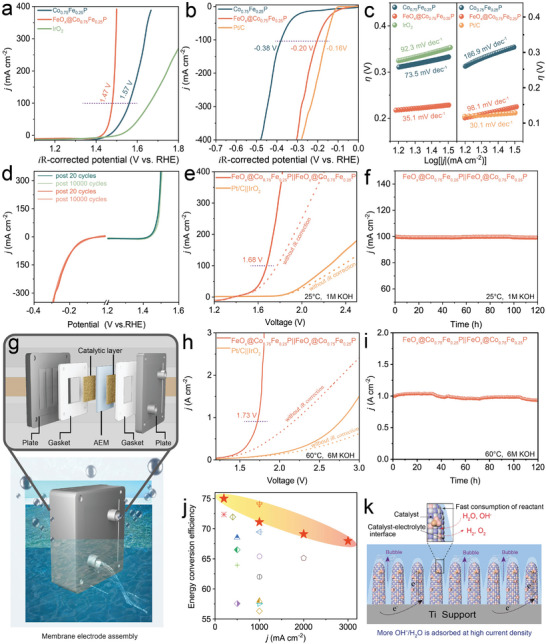
a) OER polarization curves. b) HER polarization curves. c) The corresponding Tafel plots. d) OER and HER polarization curves were measured during the ADT test. e) The polarization curves of the bifunctional FeO_x_@Co_0.75_Fe_0.25_P electrocatalyst. f) Chronopotentiometry curve of FeO_x_@Co_0.75_Fe_0.25_P. g) Schematic illustration of the MEA. h) LSV curves of the alkaline electrolyzer. i) The *i*‐*t* curves. j) comparison of energy conversion efficiency with other up‐to‐date electrocatalysts. k) Schematic illustration of the electrodes in alkaline electrolyte.

The as‐fabricated electrodes were employed as the working electrode in a 1 m KOH aqueous solution, functioning within a conventional three‐electrode electrolyzer to assess their OER and HER performances. All polarization curves were recorded from high initial potential to low potential at a scan rate of 5 mV s^−1^ to prevent signal overlap of the oxidation peak. The merits of commercial IrO_2_ catalysts loaded on Ti fiber felt were gauged for comparative insights. We engaged in polarization assessments, focusing on the OER and HER profiles of Co_0.75_Fe_0.25_P electrodes endowed with varying quotas of FeO_x_ clusters to pinpoint the zenith of catalytic activity (Figure S7, Supporting Information). As depicted in **Figure** **3**a, the optimized FeO_x_@Co_0.75_Fe_0.25_P electrode markedly transcends the catalytic outputs of both unmodified Co_0.75_Fe_0.25_P and the commercial IrO_2_ electrodes, achieving strikingly low overpotentials of a mere 240 mV at an intensity of 100 mA cm^−2^. Simultaneously, the FeO_x_@Co_0.75_Fe_0.25_P electrode requires an *i*R‐corrected 200 mV to deliver a current density of 100 mA cm^−2^ (Figure 3b), which is superior to that of Co_0.75_Fe_0.25_P (380 mV) and slightly inferior to that of commercial Pt/C (160 mV).

In addition, compared with FeO_x_ and Co_0.75_Fe_0.25_P, the HER/OER/OWS performances of FeO_x_@Co_0.75_Fe_0.25_P are greatly improved (Figure S8, Supporting Information). Besides, we derived Tafel slopes via the Butler‐Volmer formalism from the polarization curves, where the decreased Tafel slopes of FeO_x_@Co_0.75_Fe_0.25_P in both OER and HER compared to those of Co_0.75_Fe_0.25_P illustrate its improved reaction kinetics (Figure 3c). As an indicator of the best value for evaluating catalytic activity, the turnover frequencies (TOFs) are calculated as well, where the TOF values of FeO_x_@Co_0.75_Fe_0.25_P are much higher than that of Co_0.75_Fe_0.25_P and commercial IrO_2_ and Pt/C, especially at high voltage and current density (Figure S9, Supporting Information). The electrocatalytic robustness of FeO_x_@Co_0.75_Fe_0.25_P electrode for both OER and HER in alkaline electrolyte is evaluated by accelerated degradation test (ADT). Notably, no evident changes are observed in the LSV polarization curves for OER and HER after 10000 cycles, illustrating the excellent structural durability of FeO_x_@Co_0.75_Fe_0.25_P (Figure 3d).

We assemble two FeO_x_@Co_0.75_Fe_0.25_P electrodes to construct a two‐electrode system for evaluating the overall water‐splitting activity. As revealed in Figure 3e, the FeO_x_@Co_0.75_Fe_0.25_P|| FeO_x_@Co_0.75_Fe_0.25_P catalytic couple affords a current density of 100 cm^−2^ with only an applied bias of 1.67 V, which is superior to that of commercial Pt/C||IrO_2_ pair and other benchmarking catalysts for overall water‐splitting (Figure S10; Table S12, Supporting Information). In addition, the catalytic performance of FeO_x_@Co_0.75_Fe_0.25_P was superior compared to FeO_x_ and Co_0.75_Fe_0.25_P, as shown in Figure S11 (Supporting Information). Besides, the electrochemical stability for overall water splitting is investigated by long‐term chronoamperometry measurement (Figure 3f), where no obvious decay is observed after continuous operation for 120 h at a current density of 100 mA cm^−2^, confirming its robust structural stability. The H‐type electrolyzer device with the diaphragm combined with the drainage method recorded the amount of H_2_/O_2_ released at different times (Figure S12, Supporting Information), where the H_2_/O_2_ release rate is close to the theoretical value, and Faraday efficiency (FE) approaches to 100%, illustrative of the high utilization efficiency of electrons.

In addition, the assembly of the alkaline water‐splitting AEM electrolyzer is meticulously shown in Figure 3g and Figure S13 (Supporting Information). Employing bifunctional FeO_x_@Co_0.75_Fe_0.25_P as both cathode and anode, the constructed AEM electrolyzers reveal exceptional catalytic aptitude within a 1 m KOH room temperature electrolyte, most notably marked by robust activity and sustained stability when operating at the elevated current density of 1 A cm^−2^ (Figure S14, Supporting Information). Recognizing that catalysts in industrial settings typically function at higher temperatures ranging from 60 to 80 °C, evaluations of activity and stability at these temperatures become paramount for their potential industrial deployment. Operated at 60 °C in a 6 m KOH environment, the AEM electrolyzer demanded a modest cell voltage of 1.73 V to achieve a current density of 1.0 A cm^−2^, outperforming both the Co_0.75_Fe_0.25_P||Co_0.75_Fe_0.25_P and commercial IrO_2_||Pt electrode pair as evidenced in Figure 3h. This underscores the enhanced catalytic performance attributable to the FeO_x_ cluster constructed electrodes, which facilitates the adsorption capacity and rate for OH^−^/H_2_O and, in turn, modulates kinetics beyond the realm of simply non‐Faradaic processes. Besides, the endurance testing of the FeO_x_@Co_0.75_Fe_0.25_P integration unveiled consistent operation at 1.0 A cm^−2^ for 120 h with a negligible decrement in current density (Figure 3i). Moreover, no noticeable phase or morphological change of FeO_x_@Co_0.75_Fe_0.25_P after the long‐term *I*‐*t* test (Figure S15, Supporting Information) indicates its structural robustness in water‐splitting electrocatalysis. Meanwhile, the structural examination of FeO_x_@Co_0.75_Fe_0.25_P was also performed via Raman spectroscopy (Figure S16, Supporting Information), where the peak position assigned to Fe─O vibrational modes exhibited negligible shifts, illustrative of its coordination structural robustness. In addition, as an essential indicator for evaluating the energy conversion of AEM devices, the FeO_x_@Co_0.75_Fe_0.25_P alkaline AEM electrolyzer reveals dominant superiority in energy conversion efficiency relative to other benchmarking alkaline AEM electrolyzer over a wide range of current densities (Figure 3j). The enhanced performance for FeO_x_@Co_0.75_Fe_0.25_P alkaline AEM electrolyzer is illustrated in Figure 3k, where the in situ growth of FeO_x_@Co_0.75_Fe_0.25_P nanorod arrays directly onto the Ti substrate serves concurrently as a current collector and gas transport layer and markedly diminish interfacial charge transport resistance, which is confirmed by the electrochemical impedance spectroscopy (Figure S17, Supporting Information). In addition, we investigated the desorption peak of hydrogen and Gibbs free energy of the oxygen desorption, as shown in Figure S18 (Supporting Information). On the FeO_x_@Co_0.75_Fe_0.25_P surface, hydrogen and oxygen have faster desorption kinetics. The geometry of the nanorods coupled with the FeO_x_ cluster surface modification enhances rapid electron mobility and appreciably accelerates the adsorption kinetics for H_2_O/OH^−^. Meanwhile, this unique architectural configuration ensures that gas bubbles can swiftly detach from the electrode surface, particularly at high current densities, propelling the FeO_x_@Co_0.75_Fe_0.25_P electrodes to unmatched levels of activity and longevity, as well as superior energy efficiency amongst AEM electrolyzers, making them highly visible in the field of electrocatalysts (Figure S19, Supporting Information).

### 2.4 The In‐Situ Electrochemical Characterizations

**Figure 4:**
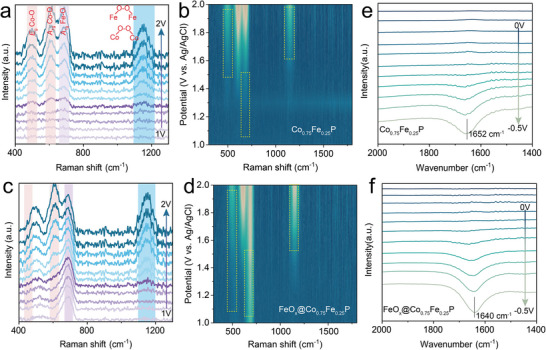
In situ surface‐enhanced Raman spectra of a, b) Co_0.75_Fe_0.25_P and c, d) FeO_x_@Co_0.75_Fe_0.25_P. In situ ATR‐FTIR spectra of e) Co_0.75_Fe_0.25_P and f) FeO_x_@Co_0.75_Fe_0.25_P.

To elucidate the influence of cluster‐induced surface charge alterations on electrocatalytic water splitting, we further conducted electrochemical characterizations based on in situ spectroscopy. The in situ electrochemical surface‐enhanced Raman spectroscopy (SERS) and shell isolated nanoparticles enhanced Raman spectroscopy (SHINERS) were employed to monitor the emergence of OER intermediates on the Co_0.75_Fe_0.25_P and FeO_x_@Co_0.75_Fe_0.25_P surfaces. The SHINERS spectra were meticulously collected at potential increments of 100 mV, charting a range from 1.0 to 2.0 V versus RHE. A noticeable absence of Raman signal was observed on the Co_0.75_Fe_0.25_P surface at potentials below 1.3 V versus RHE (**Figure** **4**a,b). This contrasted starkly with the appearance of discernible peaks at 494 and 607 cm^−1^ upon crossing the 1.4 V threshold, corresponding to the vibrational modes of CO─O and CO─OH, respectively, indicative of surface‐bound OH^−^ and inchoate CoOOH within the 1.0 m KOH electrolyte.^[^ ^42^, ^43^ ^]^ Contrastingly, the FeO_x_@Co_0.75_Fe_0.25_P surfaces revealed strong signals, indicative of OER activity well before this potential was reached (Figure 4c), suggesting exemplary catalytic behavior initiated at considerably lower voltage levels, as reinforced by the pronounced signals near 607 cm^−1^ in the contour plots (Figure 4d).^[^ ^44^, ^45^ ^]^ Furthermore, when bias was further increased to 1.3 versus RHE, the SERS detected an M─O─O─M/M─O─M adsorption peak at 1150 cm^−1^ on FeO_x_@Co_0.75_Fe_0.25_P surfaces, which is thought to be due to the oxidation of more OH^−^ adsorbed at the Fe/Co‐active site, combined with the AEM mechanism of alkaline OER. This effect was pronounced and enhanced relative to that observed on Co_0.75_Fe_0.25_P surfaces.^[^ ^46^, ^47^ ^]^ Summarily, the insights gleaned from the in situ electrochemical SERS experiments indicate that the strategic inclusion of FeO_x_ clusters promotes more efficacious surface adsorption of OH^−^ species, fundamentally underpinning the elevated OER catalytic activity of FeO_x_@Co_0.75_Fe_0.25_P under high‐current‐density. Consonantly, these experimental findings dovetail with finite element simulations of surface OH^−^ adsorption, corroborating the enhancement of catalytic functionality attributable to the heightened OH^−^ adsorption in non‐Faradaic processes.

Meanwhile, breakthroughs in HER evolution at high current densities depend on the adsorption and subsequent dissociation of H_2_O on the catalyst's surface, particularly under alkaline conditions. The HER process was probed using in situ attenuated total reflectance Fourier transform infrared (ATR‐FTIR) spectroscopy. A definitive absorption peak at 1652 cm^−1^ was resolved for Co_0.75_Fe_0.25_P, assigned to the bending vibrations of H_2_O molecules adsorbed on the catalyst surface.^[^ ^48^, ^49^ ^]^ The Co_0.75_Fe_0.25_P electrode displayed significant H_2_O adsorption peaks at even more negative potentials, reaching −0.35 V versus RHE (Figure 4e). In contrast, the intensity of this peak in FeO_x_@Co_0.75_Fe_0.25_P substantially increased at potentials of −0.25 V versus RHE, revealing pronounced adsorption of H_2_O ready to participate in the HER (Figure 4f).^[^ ^50^ ^]^ Additionally, the H_2_O adsorptive signal over FeO_x_@Co_0.75_Fe_0.25_P incurred a red shift relative to Co_0.75_Fe_0.25_P at 1640 cm^−1^, insinuating lengthened H─O─H linkages due to FeO_x_@Co_0.75_Fe_0.25_P activation, thus easing O─H bond breaking. Considering that the phosphides evolve into FeCoOOH during the catalytic process, Fe‐doped CoOOH nanorod carriers were simulated, and COMSOL finite element simulations of their surface charge densities, OH^−^ and H_2_O concentrations were performed. The simulation results are consistent with the phosphide law. Specifically, the local electric field around each cluster increased significantly, increasing the surface charge density (Figure S20, Supporting Information). The change in the local electric field induced more OH^−^ and H_2_O adsorption on the surface of the loaded cluster catalysts, the clusters adsorbed OH^−^ ions more readily, and the surface of Fe‐doped CoOOH nanorods mainly attracted H_2_O molecules, which collectively enhanced the OH_‐_/H_2_O adsorption kinetics.

### 2.5 Electronic Structural Analysis

**Figure 5:**
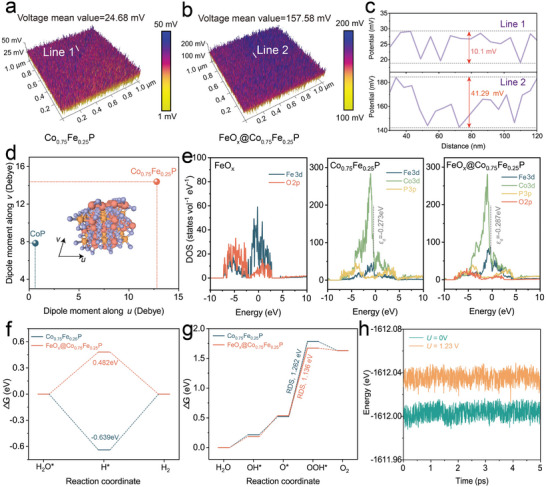
The KPFM test of a) Co_0.75_Fe_0.25_P and b) FeO_x_@Co_0.75_Fe_0.25_P. c) The corresponding potential diagram. d) Calculated dipole moment of Co_0.75_Fe_0.25_P and CoP. e) Projected density of states of FeO_x_, Co_0.75_Fe_0.25_P, and FeO_x_@Co_0.75_Fe_0.25_P. f) Gibbs free energy diagram for intermediates' evolution in HER. g) Gibbs free energy diagram for intermediates' evolution in OER. h) AIMD simulations of FeO_x_@Co_0.75_Fe_0.25_P at 0 and 1.23 V.

To illustrate the possible electronic structural change of Co_0.75_Fe_0.25_P with FeO_x_ decoration, we further conducted a deep probe using experimental characterizations and DFT calculations. The inclusion of FeO_x_ into the structure of Co_0.75_Fe_0.25_P nanorods has been observed to significantly alter their interaction with both aqueous environments and gases, boasting a superior blend of hydrophilicity and hydrophobicity.^[^ ^51^ ^]^ The FeO_x_@Co_0.75_Fe_0.25_P nanorods feature reduced contact angles, indicative of superior wettability, where the trait is especially beneficial within aqueous media, as evidenced by the observed increases in gas contact angles (Figure S21, Supporting Information). This hydrophilic‐hydrophobic confluence is advantageous for facilitating the rapid expulsion of hydrogen and oxygen gases at high current densities and ensuring the electrolyte promptly replenishes spaces vacated by escaping gas bubbles, thus sustaining swift reaction kinetics. Furthermore, the FeO_x_ clusters contribute to an amplified zeta potential and electric hysteresis polarization, implicating the nanorods' augmented surface charge density (Figure S22, Supporting Information). These findings are supported by Kelvin probe force microscopy (KPFM) measurements, which highlight a substantial variance in surface potentials with FeO_x_@Co_0.75_Fe_0.25_P exhibiting a value of 157.58 mV compared to a mere 24.68 mV for Co_0.75_Fe_0.25_P (**Figure** **5**a,b).^[^ ^52^, ^53^ ^]^ Additionally, the significant disparity of surface charges in FeO_x_@Co_0.75_Fe_0.25_P points to an induced localized polarization effect (Figure 5c). The notable surface potential, in conjunction with the noteworthy surface charge difference, plays a crucial role in regulating the adsorptive interactions of OH‐/H2O at the catalytic interface, improving catalytic efficiency. This empirical evidence aligns with the insights gleaned from finite element simulations that concentrated on the phenomenon of surface charge alterations.

In addition, we quantified the surface dipole moment of the (001) crystal plane of CoP with Fe doping (Figure 5d), where the dipole moments associated with both vectors *u* and *v* in Co_0.75_Fe_0.25_P are notably elevated, thereby reinforcing the notion that Fe integration promotes a polarization of charges at the surface interface.^[^ ^54^ ^]^ To delve deeper into the effect of the FeO_x_ cluster on the electronic structure of Co_0.75_Fe_0.25_P, Coulomb interaction‐corrected density functional theory (DFT+*U*) calculations were employed.^[^ ^55^ ^]^ As depicted in Figure 5e, the shallow trap states in FeO_x_@Co_0.75_Fe_0.25_P bear a strong resemblance to those in both FeO_x_ and Co_0.75_Fe_0.25_P, intimating that FeO_x_ incorporation affects only minor perturbations to the electronic interactions. Meanwhile, the *ε*_d_ value of Co in Co_0.75_Fe_0.25_P reveals a slight change relative to that of FeO_x_@Co_0.75_Fe_0.25_P as well, which indicates the restained electronic structure of Co_0.75_Fe_0.25_P with FeO_x_ decoration, suggesting the enhanced catalytic activity may originate from an electric field augmentation brought about by the specific configuration rather than the electronic structural changes.

Concurrently, the pivotal intermediates for the HER and OER on FeO_x_@Co_0.75_Fe_0.25_P were identified, and their Gibbs free energy changes were calculated to elucidate the rate‐determining steps (RDS) of these processes.^[^ ^56^ ^]^ The ΔG_H*_ for FeO_x_@Co_0.75_Fe_0.25_P aligns closely with the ideal thermoneutral value according to the Sabatier principle, theoretically enabling more facile hydrogen release (Figure 5f; Figure S23, Supporting Information). Similarly, Gibbs free energy reveals the *OOH formation stage as the RDS for both FeO_x_@Co_0.75_Fe_0.25_P and Co_0.75_Fe_0.25_P catalysts (Figure 5g; Figure S24, Supporting Information). Notably, the Gibbs free energy associated with the formation of ^*^OOH in FeO_x_@Co_0.75_Fe_0.25_P is merely lowered by 0.11 eV relative to Co_0.75_Fe_0.25_P, indicating that the essential uplift in OER activity is ascribed to the augmented adsorption of OH^−^ on the catalyst's surface rather than the energetic profile of reaction intermediates (Figure S25, Supporting Information). Besides, in assessing the structural stability of the cluster‐modified catalysts, we employed ab initio molecular dynamics (AIMD) simulations for FeO_x_@Co_0.75_Fe_0.25_P. Impressively, the configurations of FeO_x_@Co_0.75_Fe_0.25_P sustained their original geometry after 5 picosecond simulations at both 0 and 1.23 V (Figure 5h), attesting to the formidable stability of the modified catalyst.

### 2.6 Valence State and Coordination Structural Analysis

**Figure 6:**
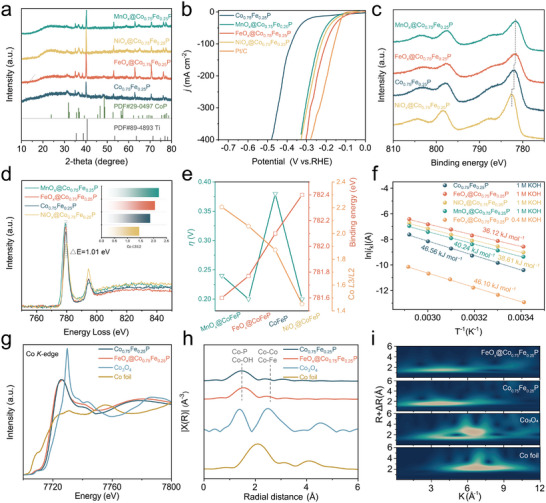
a) XRD pattern. b) HER polarization curves. c) The Co 2p XPS spectra results. d) The EELS of Co *L*_3_ edges. e) The Comparison of overpotential at −100 mA cm^−2^ and electron binding energy, *L*_3_/*L*_2_. f) Arrhenius plot of the kinetic current. g) Co K‐edge XANES spectra. The corresponding FT‐ h) EXAFS spectra and i) WT‐EXAFS plots.

To verify whether the electrocatalytic activity can be regulated by manipulating localized interfacial charge, we thereby synthesized a suite of catalysts by anchoring heterogeneous metal oxide clusters, namely MnO_x_ and NiO_x_, onto Co_0.75_Fe_0.25_P nanorods. Adopting a similar synthesis approach as that employed in FeO_x_@Co_0.75_Fe_0.25_P, MnO_x,_ and NiO_x_ clusters were successively anchored to Co_0.75_Fe_0.25_P nanorods employing impregnation, where XRD plots confirmed that the underlying crystal structure of Co_0.75_Fe_0.25_P nanorods is unchanged (**Figure** **6**a). Meanwhile, the HRTEM images evidence that clusters with an average size of 5 nm anchor on the nanorods, and EDX elemental mappings further confirm the homogenous distribution of elemental composition in MnO_x_@Co_0.75_Fe_0.25_P and NiO_x_@Co_0.75_Fe_0.25_P (Figure S26, Supporting Information). We compared the HER and OER performances of these cluster‐modified nanorods with commercial Pt/C catalysts. Interestingly, the modified catalysts outperformed the pure Co_0.75_Fe_0.25_P nanorods (Figure 6b; Figure S27, Supporting Information).

To probe the underlying reasons, we scrutinized the electronic structure using XPS spectra to unveil the modulations in the cobalt valence state concomitant with the introduction of different metal oxide clusters (Figure 6c), where the binding energy of Co atoms in host Co_0.75_Fe_0.25_P appears swings with MnO_x_ and NiO_x_ decoration. Additionally, the electron energy loss spectroscopy (EELS) further illustrated these alterations, with redshifts observed in the MnO_x_@Co_0.75_Fe_0.25_P and FeO_x_@Co_0.75_Fe_0.25_P, as well as a blue‐shift in the NiO_x_@ Co_0.75_Fe_0.25_P white line, proposing a lower cobalt valence state in the former two and a heightened state in the latter (Figure 6d).^[^ ^57^, ^58^ ^]^ The edge ratio of Co *L*3/*L*2, a sensitive proxy for Co valence state, aligned with these findings from the XPS spectra, indicating the promoted electrocatalytic performance unrelated to the valence of the active center species (Figure 6e). These outcomes suggest that structural clusters engender a catalytic performance enhancement, a phenomenon detached from the valency of the active centers, thereby challenging the established principles guiding traditional catalyst design, predominantly predicated on valence state or charge density adjustments of the central atom. In addition, the activation energy (*E*_a_) associated with the cluster‐anchored catalyst is notably diminished. To curtail the effect of OH^−^ concentration within the electrolyte on the adsorption dynamics of the surface compact layer, a dilute electrolyte solution (0.4 m) was deployed to ascertain the *E*_a_ of FeO_x_@Co_0.75_Fe_0.25_P. The *E*_a_ of FeO_x_@Co_0.75_Fe_0.25_P within this lower concentration milieu is distinctly decreased and approaches that of pristine Co_0.75_Fe_0.25_P, underscoring the paramountcy of the interfacial field effect that is rooted in the catalyst's topological architecture as a primary determinant of catalytic efficiency (Figure 6f).

The local coordination environment and electronic structure of the catalysts were further elucidated through X‐ray absorption spectroscopy (XAS). After 100 cycles of cyclic voltammetry, the absorption edge energy for FeO_x_@Co_0.75_Fe_0.25_P and Co_0.75_Fe_0.25_P was positioned between that of metallic Fe and Fe_2_O_3_, closely approximating the latter, which implies that the iron atoms in the catalyst exhibit a valence state proximal to +3, with Fe in FeO_x_ showing a marginally higher valence than in Co_0.75_Fe_0.25_P (Figure S28, Supporting Information). Fourier‐transformed extended X‐ray absorption fine structure (FT‐EXAFS) spectra revealed a principal peak at ≈ 1.5 Å, attributable to Fe─O or Fe─P coordination, with an increased peak intensity for FeO_x_@Co_0.75_Fe_0.25_P, indicative of the presence of Fe─O and Fe─P scattering paths and affirming the successful incorporation of FeO_x_ clusters (Figures S29–S32, Supporting Information).^[^ ^59^ ^]^ In cobalt K‐edge XAS analysis, the white line features for both FeO_x_@Co_0.75_Fe_0.25_P and Co_0.75_Fe_0.25_P post‐electrochemical cycling seated between measurements of cobalt metal and Co_3_O_4_ indicate a cobalt valence state intermediate between 0 and +8/3(Figure 6g).^[^ ^60^ ^]^ The cobalt valence in FeO_x_@Co_0.75_Fe_0.25_P is slightly reduced compared to that in Co_0.75_Fe_0.25_P alone. Besides, the FT‐EXAFS also unveiled that the incorporation of FeO_x_ clusters did not notably alter the Co_0.75_Fe_0.25_P bonding environment (Figure 6h). In addition, the wavelet transforms corroborated this with pronounced signals observed at ≈3.5 Å^−1^ in *k*‐space and ≈1.5 Å in *R*‐space for both FeO_x_@Co_0.75_Fe_0.25_P and Co_0.75_Fe_0.25_P, further confirming the structural integrity of the Co_0.75_Fe_0.25_P matrix upon FeO_x_ loading (Figure 6i).

Cumulatively, this evidence indicates an enhancement in the electrocatalytic activity that arises not from electronic or structural modifications within the material itself but rather from an interfacial charge enrichment attributable to the interfacial electric field manipulation. To explore the generalizability of this mechanistic insight, we extended our study to include nanosheet catalysts with varying cluster loadings (Figures S33 and S34, Supporting Information), which comparably exhibited improved HER/OER activities relative to the bare Co_0.75_Fe_0.25_P nanosheets (Figure S35, Supporting Information). Finite element analysis further elucidated increased surface charge density following cluster augmentation, stimulating higher adsorption rates of OH^−^/H_2_O onto the material's surface, which was decisive for the catalytic activity boost (Figure S36, Supporting Information). This work presents a compelling case for interfacial charge enrichment through strategic cluster decoration as an innovative strategy for the future of electrocatalyst design, potentially unlocking new frontiers in the quest for efficient and robust catalyst systems.

## 3 Conclusion

In this study, we proposed an interfacial electric field regulation strategy by manipulating the topological structure of electrocatalyst. Specifically, the electrochemical characterizations and theoretical calculations suggest that the enhanced electrocatalytic water‐splitting performance in FeO_x_@Co_0.75_Fe_0.25_P mainly aroused from the enhanced adsorptive phenomena fostered by the bolstered electric field rather than alterations in the valence states and coordination geometries of the central active sites, which highlights the importance of the non‐Faradic process of OH^−^/H_2_O absorption in alkaline electrolysis. These findings challenge traditional regulating strategies centered on active sites. Based on the insights and principles, our rationally designed FeO_x_@Co_0.75_Fe_0.25_P electrocatalyst reveals superior performances in the two‐electrode and MEA electrolyzer. Specifically, in the MEA electrolyzer, the 1.0 A cm^−2^ can be achieved at a cell voltage of only 1.73 V for the FeO_x_@Co_0.75_Fe_0.25_P electrode pairs at 60 °C and 6 m KOH industrial environment with an energy conversion efficiency of 71%, which outperforms most of the benchmarking overall alkaline water‐splitting electrocatalysts. Moreover, the manifestation of analogical enhancements in Co_0.75_Fe_0.25_P nanosheets anchored with FeO_x_ clusters further confirms the widespread applicability of this strategy. This study delineates a persuasive argument for adopting interfacial charge enhancement and paves the way for constructing efficient alkaline electrolysis by regulating the non‐Faradic process.

## 4 Experimental Section

### Synthesis of Co_0.75_Fe_0.25_P Electrode

Ti fiber felt (2 × 4 cm^2^) was immersed in 0.5 m HCl solution for 30 min, and it was sequentially cleaned with ethanol and deionized water and left to dry overnight. Then, 3 mmol Co(NO_3_)_2_·6H_2_O, 1 mmol Fe(NO_3_)_2_·9H_2_O, 7 mmol NH_4_F, and 13.5 mmol CO(NH_2_)_2_ were dissolved in 35 mL deionized water under magnetic stirring for 30 min.^[^ ^61^ ^]^ Subsequently, a piece of the previously treated Ti fiber felt and the solution above was placed into a 50 mL autoclave and maintained at 120 °C for 6 h. Upon completion of the reaction, the Ti fiber felt was subjected to multiple washings with ethanol and deionized water and dried at 80 °C. The Ti fiber felt was further annealed at 400 °C for 1 h with a heating rate of 2 °C min^−1^ in an Ar atmosphere with 0.6 g NaH_2_PO_2_ positioned at the furnace upstream. The sample was denoted as Co_0.75_Fe_0.25_P. Utilizing the same procedure but with varying proportions of metal salt precursors, four additional distinct catalysts, namely CoP, Co_0.875_Fe_0.125_P, Co_0.625_Fe_0.375_P, and Co_0.5_Fe_0.5_P, were obtained.

### Synthesis of FeO_x_@Co_0.75_Fe_0.25_P Electrode

The Co_0.75_Fe_0.25_P was subjected to impregnation in a mixture solution of 4 mL of ethanol and 200 µL of FeCl_3_ solution (50 mg mL^−1^) at room temperature for 4 h. Following this process, the electrodes were transferred into a tube furnace and thermal‐treated at 400 °C for 2 h under the Ar atmosphere. After cooling to room temperature, the electrodes were obtained and denoted as FeO_x_@Co_0.75_Fe_0.25_P. Using the same method but with different types of metal salt precursors, two other distinct catalysts were obtained, namely MnO_x_@Co_0.75_Fe_0.25_P and NiO_x_@Co_0.75_Fe_0.25_P.

### Synthesis of Cluster Anchored Co_0.75_Fe_0.25_P Nanosheet Electrode

Typically, 3 mmol Co(NO_3_)_2_·6H_2_O, 1 mmol Fe(NO_3_)_2_·9H_2_O, and 15 mmol C_6_H_12_N_4_ were dissolved in 40 mL deionized water under magnetic stirring for 30 min. Subsequently, a piece of the previously treated Ti fiber felt and the solution above was placed into a 50 mL autoclave and maintained at 100 °C for 10 h.^[^ ^62^ ^]^ Subsequently, the Ti fiber felt was subjected to multiple washings with ethanol and deionized water and dried at 80 °C. The Ti fiber felt was further annealed at 400 °C for 1 h with a heating rate of 2 °C min^−1^ in an Ar atmosphere with 0.6 g NaH_2_PO_2_ positioned at the furnace upstream. The sample was denoted as a Co_0.75_Fe_0.25_P sheet. Then, the Co_0.75_Fe_0.25_P nanosheet was subjected to impregnation in a solution of 4 mL of ethanol and 200 µL of Fe(NO_3_)_3_ or Mn(NO_3_)_2_ or Ni(NO_3_)_2_ (50 mg mL^−1^) at room temperature for 4 h. Following this process, the electrodes were transferred into a tube furnace and thermal‐treated at 400 °C for 2 h under the Ar atmosphere. After cooling to room temperature, the electrodes were obtained and denoted as FeO_x_@Co_0.75_Fe_0.25_P nanosheet, MnO_x_@Co_0.75_Fe_0.25_P nanosheet, and NiO_x_@Co_0.75_Fe_0.25_P nanosheet.

## Conflict of Interest

The authors declare no conflict of interest.

## Author Contributions

Y.X. supervised the project. N.W. performed all sample synthesis and characterizations, and Y.X. performed all the calculations. H.W. provided in situ electrochemical analysis, Q.L. provided COMSOL simulation analysis and K.S. provided spherical aberration electron microscopy testing. X.J. and D.C. discussed the experimental and theoretical results. N.W. and Y.X. wrote this manuscript, and all authors contributed to the overall scientific interpretation and revised this paper.

## Supporting information

Supporting Information

## Data Availability

The data that support the findings of this study are available from the corresponding author upon reasonable request.
