# Supplementary material for: Field‐Effect Enhancement of Non‐Faradaic Processes at Interfaces Governs Electrocatalytic Water Splitting Activity
Source: Adv Sci (Weinh). 2024 Jun 27;11(33):2403206. doi: 10.1002/advs.202403206 (PMC11434135; doi:10.1002/advs.202403206)
Supplement: Supplementary file 1 — Supporting Information [file ADVS-11-2403206-s001.docx]

Supporting Information

**Field-Effect Enhancement of Non-Faradaic Processes at**

**Interfaces Governs Electrocatalytic Water Splitting Activity**

*Ning Wen, Haihua Wang, Qilu Liu, Kepeng Song, Xiuling Jiao, Yuguo Xia*and Dairong Chen**

N. Wen, H. Wang , H. Li, K. Song, Prof X. Jiao, Dr. Y. Xia, Prof. D. Chen
National Engineering Research Center for Colloidal Materials

School of Chemistry and Chemical Engineering

Shandong University

Jinan, Shandong 250100, P. R. China

E-mail: *xyg@sdu.edu.cn; cdr@sdu.edu.cn*

Q. Liu,
State Key Laboratory of Crystal Materials

Shandong University

Jinan, Shandong, China, 250100

**Methods**

**Physicochemical Characterizations**

X-ray diffraction (XRD) measurements were performed utilizing a D/Max 2200pc diffractometer equipped with Cu Kα radiation (λ= 1.5418 Å). To characterize the morphology of the samples, secondary electron images were obtained using a scanning electron microscope (SEM) from Hitachi, model SU8010, and aberration-corrected transmission electron microscopy (TEM) employing a JEM-ARM200F instrument. The elemental compositions and bonding information were determined using the X-ray photoelectron spectroscopy (XPS) technique with 30.0 eV pass energy and an Al Kα line excitation source (Thermo Kalpha). Furthermore, the atomic phase of the materials was characterized through high-angle annular dark-field (HAADF) imaging (FEI Theims Z). Inductively coupled plasma-mass spectrometry (ICP-MS) measurements were conducted on a PerkinElmer 8300 instrument to determine the catalysts' composition. The nanoparticles' surface and zeta potential were analyzed using a nanoparticle size and zeta potential analyzer (Malvern). Additionally, the electric polarization curve of the catalyst was measured using a ferroelectric analyzer (ACIX TF2000E). Electrochemical processes were characterized by *in*-*situ* electrochemical infrared (ThermoFisher Scientific iS50 FT-IR) and *in*-*situ* electrochemical Raman (HORIBA, LabRAM Odyssey).

**XAS analysis**

The X-ray absorption fine structure (EXAFS) spectra were collected at the Shanghai Synchrotron Radiation Facility, where the storage rings of BSRF operated at 2.5 GeV with a maximum current of 250 mA. Si (111) double-crystal monochromator was employed for data collection in transmission mode, and an ionization chamber was utilized. All spectra were acquired under ambient conditions. Subsequently, the obtained EXAFS data were processed following standard procedures using the ATHENA module implemented in the IFEFFIT software packages. The quantitative structural parameters around central atoms were obtained by performing least-squares curve parameter fitting using the ARTEMIS module of the IFEFFIT software packages.^[1]^

**Electrocatalytic measurements**

Electrochemical experiments were conducted in an electrolytic cell using a standard three-electrode system on a CHI760E electrochemical workstation. The working electrodes utilized in these experiments were the as-prepared samples, with an approximate catalyst loading of ≈3 mg cm^–2^. A graphite rod was employed for the counter electrode, while a Hg/HgO electrode (1.0 M KOH) served as the reference electrode. Before the measurements, the electrolyte was saturated with O_2_ to ensure consistent conditions. Both linear sweep voltammetry and cyclic voltammetry were performed at a sweep speed of 5 mVs^-1^. To calibrate the measured potentials, they were referenced to the reversible hydrogen electrode (RHE) using the equation: *E*_RHE_ = *E*_Hg/HgO_ + 0.098 + 0.059 × pH. Electrochemical impedance spectroscopy (EIS) spectra were recorded at the open-circuit potential, covering a frequency range from 1 MHz to 0.01 Hz. The high current density was tested using programmable Itechi current recording.

**Finite element analysis**

We employed the finite element analysis capabilities of COMSOL Multiphysics software to simulate a two-dimensional model using the Electric Currents and Transport of Diluted Species modules.^[2]^ The focus of the model was on a two-dimensional axisymmetric catalyst, with a particular emphasis on the catalytic reactions occurring on the electrode surface. The model was discretized using free tetrahedral meshes. To ensure computational accuracy, a relative tolerance of 0.01 was set for the steady-state solver.

The simulation of the electric field was conducted using potential difference, with the calculation formula as follows:

$E=-\nabla V$ (S1)

Furthermore, the charge density was computed using Gauss's Law:

$D=\varepsilon_{0}\varepsilon_{\text{r}}E$ (S2)

Where ε_0_ represents the permittivity of free space and ε_r_ is the material's relative permittivity. In our model, the permittivity of the electrolyte was set to 78.5, and that of the electrode was set to 1. Additionally, the conductivity of CoP material was chosen as 3.71 S cm^-1^, and the conductivity of the electrolyte was set at 0.1 S cm^-1^ .^[4]^

The double layer at the electrode interface was modeled using the Gouy-Chapman-Stern model, which comprises both a Helmholtz layer and a diffusion layer. The Helmholtz layer is composed of OH^-^/H_2_O molecules adsorbed on the electrode surface, serving as reactants for OER/HER. The diffusion layer, on the other hand, consists of cations and anions present in the electrolyte. The Poisson-Nernst-Planck equation was solved under the condition of dynamic equilibrium between electrostatic and diffusive forces, represented by the following formulations:

$\nabla^{2}V=\left\{\begin{matrix} 0 d\text{＜}d_{\mathrm{H}} \\ (C_{\mathrm{K}}-C_{\mathrm{OH}})F d\text{＞}d_{\mathrm{H}} \end{matrix}\right\}$ (S3)

(S4)

Here, d denotes the distance from the electrode surface to the electrolyte, and d_H_ is the thickness of the Helmholtz layer, assumed to be the radius of the hydroxide ion (0.137 nm). *c*_i_ with i∈{K^+^, OH^-^}represent the concentrations of potassium and hydroxide ions, respectively, while z_i_ indicates their charge numbers, and e is the elementary charge. k_B_ is the Boltzmann constant, with an absolute temperature of T = 297.3 K. The diffusion coefficients D for potassium ions, hydroxide ions, and protons in water are 2.14×10^-9^ m^2^ s^-1^, 1.9×10^-9^m^2^ s^-1^, and 7.10×10^-9^ m^2^ s^-1^, respectively. To ensure the accuracy of the model, optimizations of cell size, mesh type, and density were carried out, similar to the methodologies described in the literature^36^.

**DFT calculations**

Spin-polarized density functional theory (DFT) computations were carried out employing the projected augmented wave (PAW) methodology within the Vienna ab initio simulation package (VASP).^[3]^ Exchange-correction potentials were addressed via the generalized gradient approximation (GGA) endowed by the Perdew-Burke-Ernzerhof (PBE) functionals. Electronic wave functions were described by a plane wave basis set, truncated at an energy cutoff of 520 eV. To adequately represent the strong on-site Coulomb interactions of Co and Fe atoms, we incorporated effective *U*-*J* values of 3.50 and 2.56 eV, ^[4]^ respectively. The lower boundary atoms were terminated with H and pseudo-potential 0.5H atoms to mitigate the effects of dangling bonds. The *k*-point sampling for Brillouin zone integration conformed to the Monkhorst-Pack scheme, with a Kmesh resolution maintained at 0.03 (2π/Å). Convergence thresholds for electronic relaxations and structural optimizations were set to 10^-5^ eV and 0.02 eV Å^-1^, respectively. The structural rigidity of FeO_x_@Co_0.75_Fe_0.25_P under thermal conditions was probed through ab initio molecular dynamics (AIMD) simulations^64^. The simulations were realized in the canonical ensemble (NVT), maintained by a Nosé-Hoover thermostat at a physiological temperature of 300 K. A temporal discretization of 1 fs and a total simulation span of 5 ps was chosen to ensure thermodynamic equilibrium and mitigation of transient artifacts. Besides, the solvent effects on the crucial intermediates involved in the HER and OER evolutions have been considered by using the Poisson-Boltzmann implicit solvent model, where the dielectric constant (*ε*) is taken as 80 for water.

**Surface structural determination for Co_0.75_Fe_0.25_P**

The chemical potentials represent the growth conditions of atoms, which must be carefully treated to investigate the relative stability of introduced defects. Under thermal equilibrium growth conditions, CoP should satisfy eq S5. Moreover, precipitation of secondary phases such as FeP and CoFeP (orthorhombic, *a*=5.712Å, *b*=3.527Å, *c*=6.525Å ) should be excluded. Simultaneously, the chemical potential of each element must not be larger than the corresponding chemical potential of the bulk element. In equations, all conditions can be summarized as follows.

$\Delta\mu_{\text{Co}}+\Delta\mu_{\text{p}}=H_{\mathrm{CoP}}^{\mathrm{f}}$ (S5)

$\Delta\mu_{\text{Fe}}+\Delta\mu_{\text{p}}\leq H_{\mathrm{FeP}}^{\mathrm{f}}$ (S6)

$\Delta\mu_{\text{Fe}}+\Delta\mu_{\text{Co}}+\Delta\mu_{\text{p}}\leq H_{\mathrm{CoFeP}}^{\mathrm{f}}$ (S7)

$\Delta\mu_{\mathrm{Fe}}\leq0 ;\Delta\mu_{\mathrm{Co}}\leq0 ;\Delta\mu_{\mathrm{P}}\leq0$ (S8)

According to the equations described above, two conditions should be included: the Fe-rich environment and the P-rich environment. In the Fe-rich environment, the upper chemical potential boundary of the Fe element is determined to be $\Delta\mu_{\text{Fe}}\text{=}H_{\mathrm{CoFeP}}^{\mathrm{f}}-H_{\mathrm{CoP}}^{\mathrm{f}}$; therefore, the chemical potential of P and Co correspond to$\Delta\mu_{\mathrm{P}}\text{=}H_{\mathrm{FeP}}^{\mathrm{f}}-\text{(}H_{\mathrm{CoFeP}}^{\mathrm{f}} -H_{\mathrm{CoP}}^{\mathrm{f}}\text{)}$and $\Delta\mu_{\mathrm{Co}}\text{=}H_{\mathrm{CoFeP}}^{\mathrm{f}} -H_{\mathrm{FeP}}^{\mathrm{f}}$(Δ*μ*_Fe_=-0.492 eV, Δ*μ*_Co_=-0.451 eV, Δ*μ*_P_=-0.747 eV). Meanwhile, the upper potential boundary of the P element is determined to be $\Delta\mu_{\mathrm{P}}\text{=}0$; therefore, the chemical potential Co and Fe correspond to$\Delta\mu_{\mathrm{Co}}\text{=}H_{\mathrm{CoP}}^{\mathrm{f}}$and $\Delta\mu_{\text{Fe}}\text{=}H_{\mathrm{CoFeP}}^{\mathrm{f}}- H_{\mathrm{CoP}}^{\mathrm{f}}$(Δ*μ*_Fe_=-1.239 eV, Δ*μ*_Co_=-1.198 eV, Δ*μ*_P_=0 eV).

Given the negative charge characteristic of Co_0.75_Fe_0.25_P, its surface should be considered to be a P-rich environment. Therefore, two slab models were constructed, and the surface energy of (001) crystal plane of CoFeP can be expressed as:

$\gamma_{001\_\mathrm{CoP}}\text{=}\frac{1}{2A}(E_{\mathrm{s}\text{lab}}^{\mathrm{relax}}-8\mu_{\text{Co}}-2\mu_{\mathrm{Fe}}-10\mu_{\mathrm{P}})$ (S9)

$\gamma_{100\_CoFeP}\text{=}\frac{1}{2A}(E_{\mathrm{s}\text{lab}}^{\mathrm{relax}}-7\mu_{\text{Co}}-3\mu_{\mathrm{Fe}}-10\mu_{\mathrm{P}})$ (S10)

The surface energies of the (001) crystal plane truncated with CoP and CoFeP atoms are calculated to be 1.044 J m^-2^ and 1.636 J m^-2^, respectively, where the (001) crystal plane truncated with the CoP atoms reveals lower surface energy. Thus, the following surface structural analysis and the construction of FeO_x_*@*Co_0.75_Fe_0.25_P heterojunction are based on the (001) crystal plane truncated with the CoP atoms.

**HER mechanism calculation**

The key reaction steps of HER under alkaline media can be described as:

$H_{2}O+\text{e}^{\text{-}}+*\to H*+OH^{-}$ (S11)

$2H*\to*+H_{2}\uparrow$ (S12)

Where the asterisk (*) stands for the active site on the catalyst, while H* represents the intermediates during the HER revolution.

Herein, Gibbs' free energy in the HER process was corrected at 298.15K, and taking ∆*G*_H*_ as an example, ∆*G*_H*_ can be evaluated by Equation S13:

$\Delta G_{H*}=\Delta E_{H*}+\Delta E{}_{ZPE}-T\Delta S_{H}$ (S13)

Where ∆*E*_H*_, ∆*E_ZPE_*, and *T*∆*S*_H_ represent the differences in hydrogen adsorption energy, zero-point energy, and the entropy between adsorbed hydrogen and hydrogen in the gas phase, respectively.

**OER mechanism calculation**

The OER process proceeding through a four-electron pathway in alkaline media is summarized as the following elementary steps:

$*+\text{OH}^{\text{-}}\to\mathrm{OH}*+\mathrm{e}^{-}$ (S14)

$\mathrm{OH}*+\mathrm{OH}^{-}\to\mathrm{O}*+\mathrm{H}_{2}\mathrm{O}+\mathrm{e}^{-}$ (S15)

$\mathrm{O}*+\mathrm{OH}^{-}\to\mathrm{OOH}*+\mathrm{e}^{-}$ (S16)

$\mathrm{OOH}*+\mathrm{OH}^{-}\to*+\mathrm{O}_{2}+\mathrm{H}_{2}\mathrm{O}+\mathrm{e}^{-}$ (S17)

Where the asterisk (*) stands for the active site on the catalyst, while O*, OH*, and OOH* represent the intermediates during the OER evolution.

For each step, the reaction Gibbs free energy (∆G) is defined as the difference between free energies of the initial and final states and is given by the expression,

$\Delta G=\Delta E+\Delta ZPE-T\Delta S-eU$ (S18)

Where ∆*E* is the reaction energy of the reaction and product molecules absorbed on the catalyst surface obtained from DFT calculations, ∆ZPE is the difference of zero-point energy, *T* equals 298.15 K, ∆*S* is the entropy change, and *U* is the potential applied at the electrode. The energies of H_2_O(l) and H_2_(g) are referenced in Nørskov's work,^[5]^ and frequencies of adsorbed species, which are calculated for the zero-point energy (ZPE) corrections, are supplemented in Table S10-S11.

The overpotential (*η*_OER_) for OER can be obtained by eq S19.

$\eta_{\mathrm{OER}}=\frac{\max\{\Delta G_{1},\Delta G_{2},\Delta G_{3},\Delta G_{4}\}}{e}-1.23 V$ (S19)

Where ∆G_1_, ∆G_2_, ∆G_3_, and ∆G_4_ are the free energies of reactions, respectively.


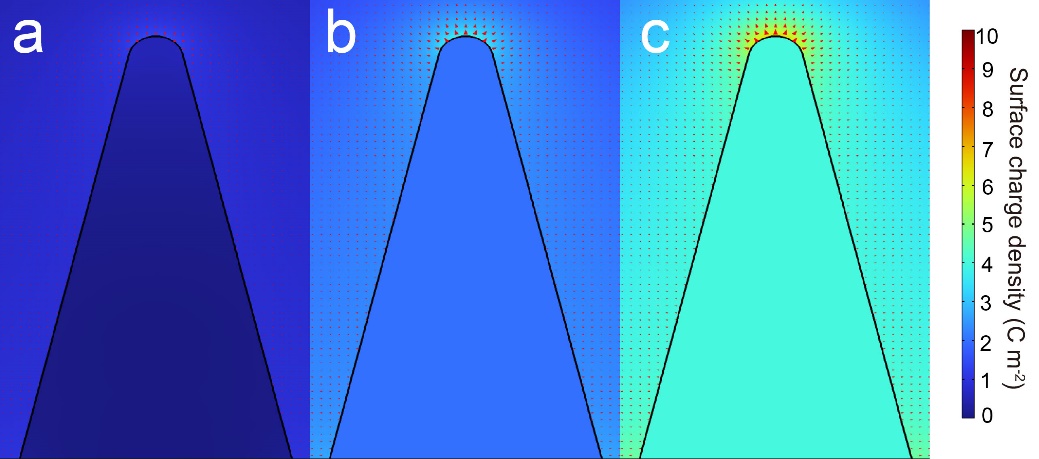


**Figure S1.** Charge density distribution on the surface of (a) CoP, (b) Co_0.875_Fe_0.125_P, and (c) Co_0.75_Fe_0.25_P.


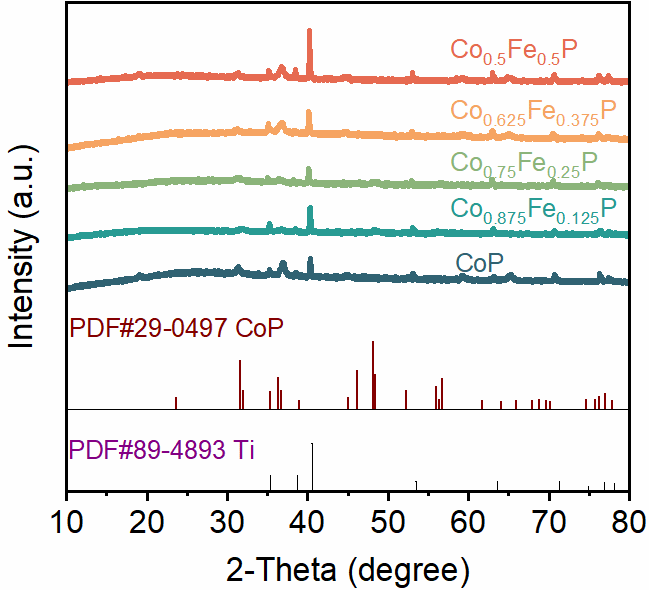


**Figure S2.** XRD pattern of the prepared Co_x_Fe_1-x_P catalyst. As the ratio of Co and Fe changes, the phase of the catalyst remains unchanged and continues to correspond with the standard card of CoP (PDF#29-0497).


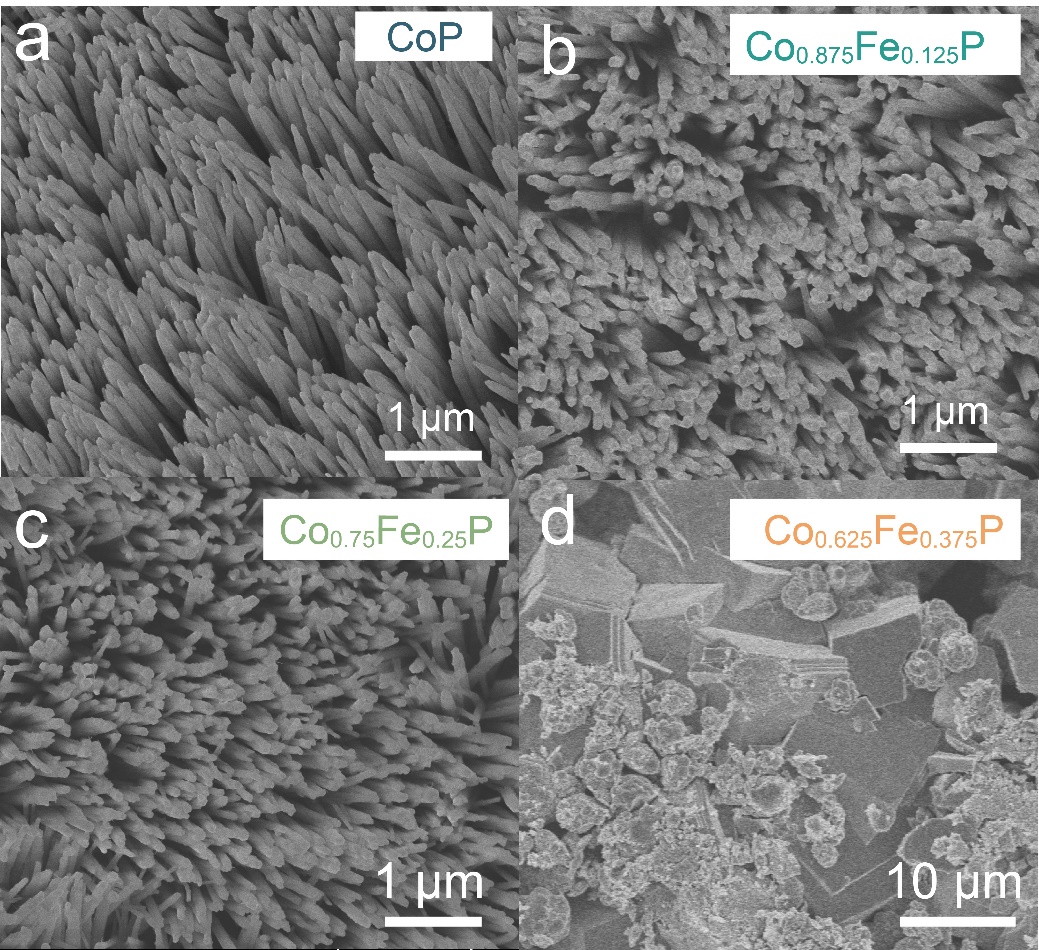


**Figure S3.** SEM of the prepared Co_x_Fe_1-x_P catalyst.


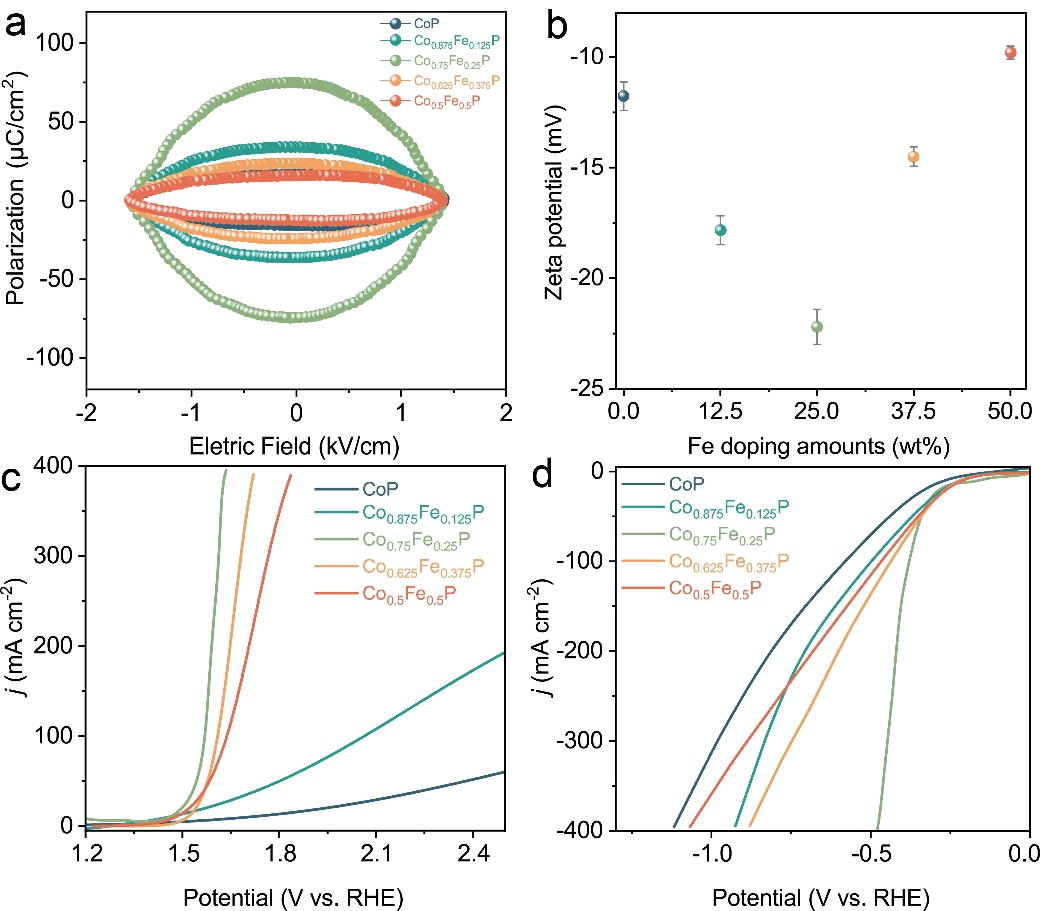


**Figure S4.** (a) Electric polarization curve. (b) Zeta potential of Co_x_Fe_1-x_P. (c) OER polarization curves. (d) HER polarization curves of Co_x_Fe_1-x_P.


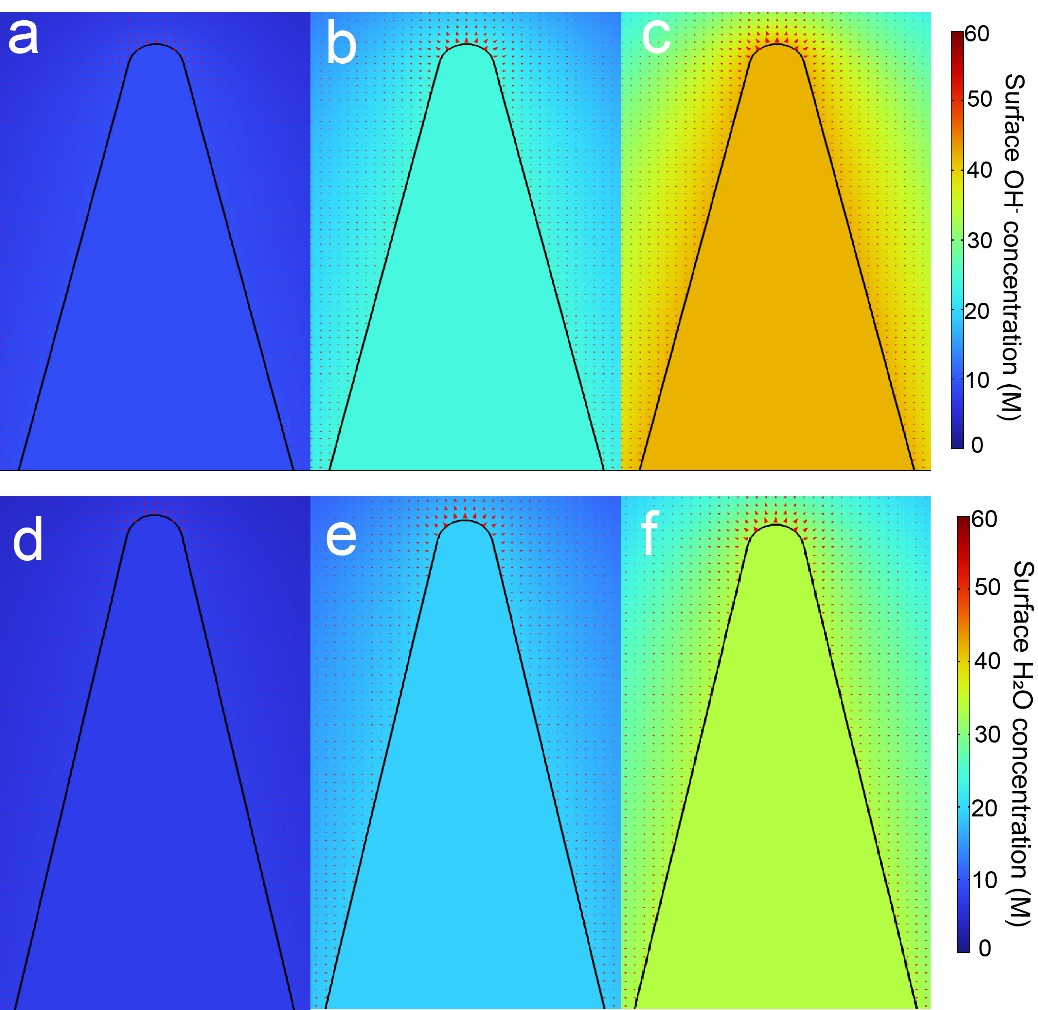


**Figure S5.** Surface OH^−^density distribution on the electrode surface of (a) CoP, (b) Co_0.875_Fe_0.125_P, (c) Co_0.75_Fe_0.25_P. Surface H_2_O density distribution on the electrode surface of (d) CoP, (e) Co_0.875_Fe_0.125_P, (f) Co_0.75_Fe_0.25_P. The surface of Co_0.75_Fe_0.25_P adsorbs the most H_2_O and OH^-^, which is consistent with surface charge density. Therefore, Co_0.75_Fe_0.25_P exhibits the best catalytic activity.


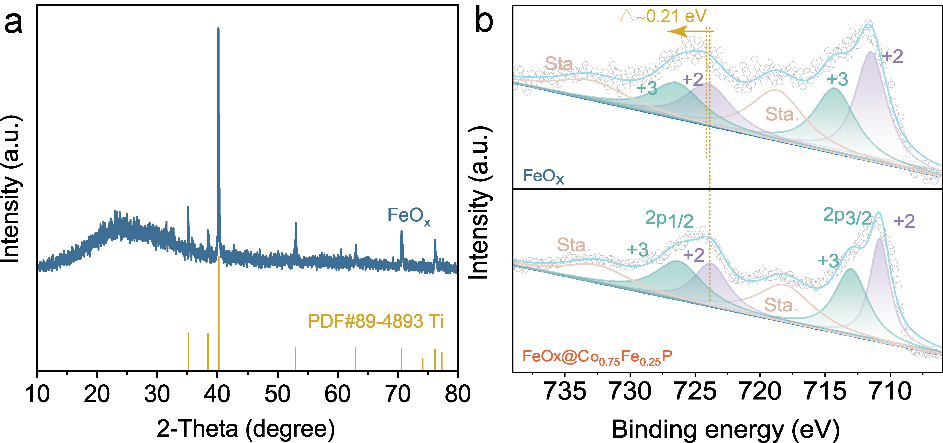


**Figure S6**. a) XRD patterns. b) XPS of FeO_x_ and FeO_x_@Co_0.75_Fe_0.25_P.


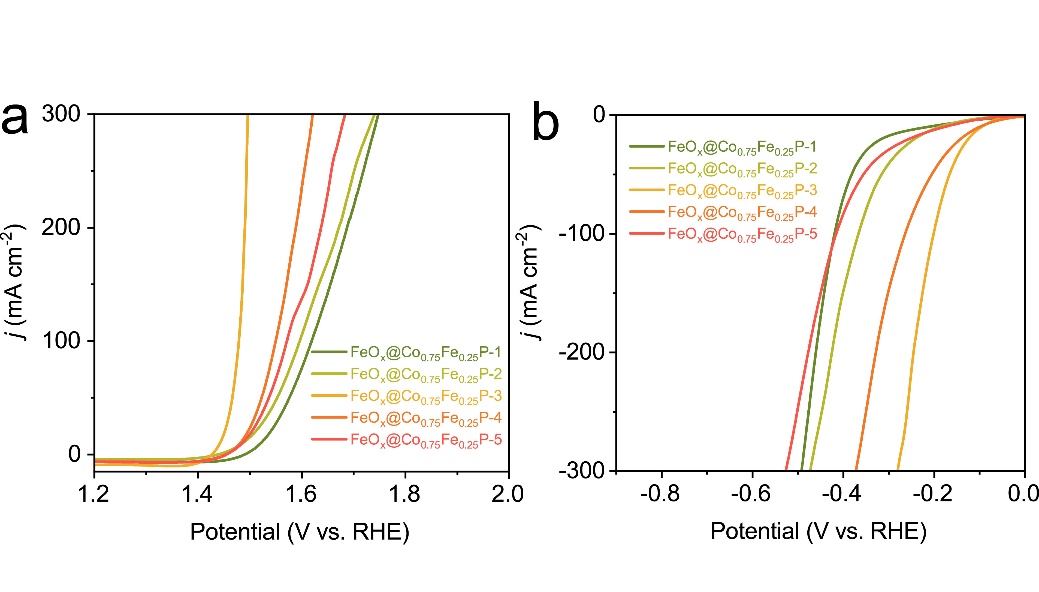


**Figure S7.** (a) OER polarization curves. (b) HER polarization curves.


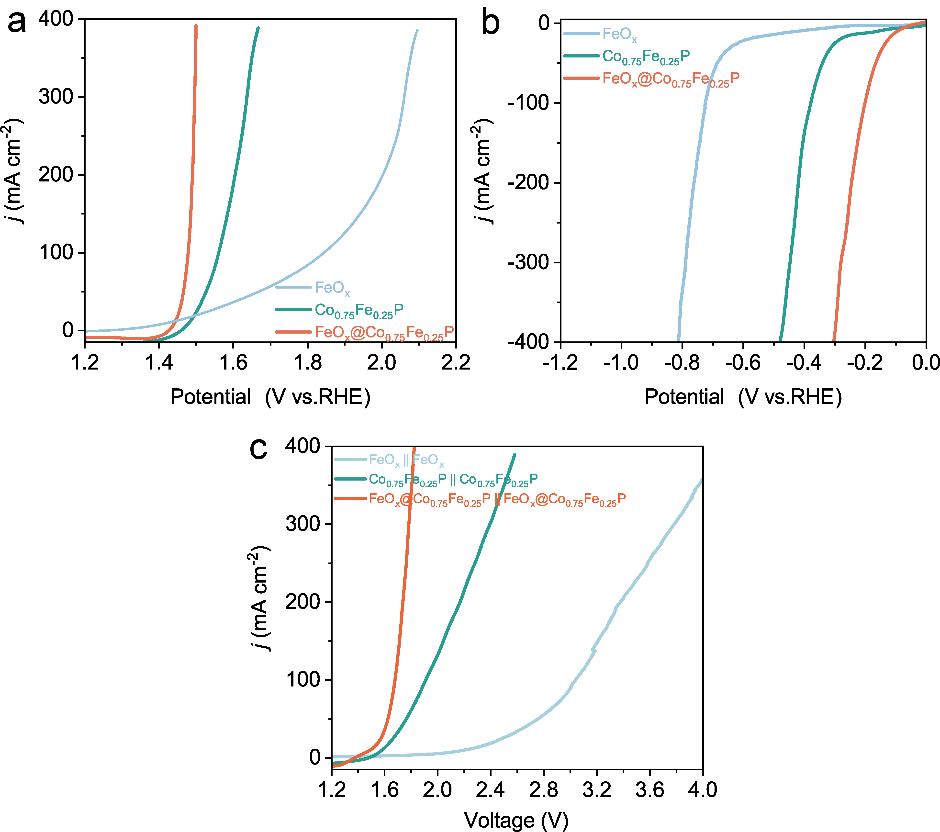


**Figure S8**. a) OER polarization curves. b) HER polarization curves. c) LSV curves of the alkaline electrolyzer.


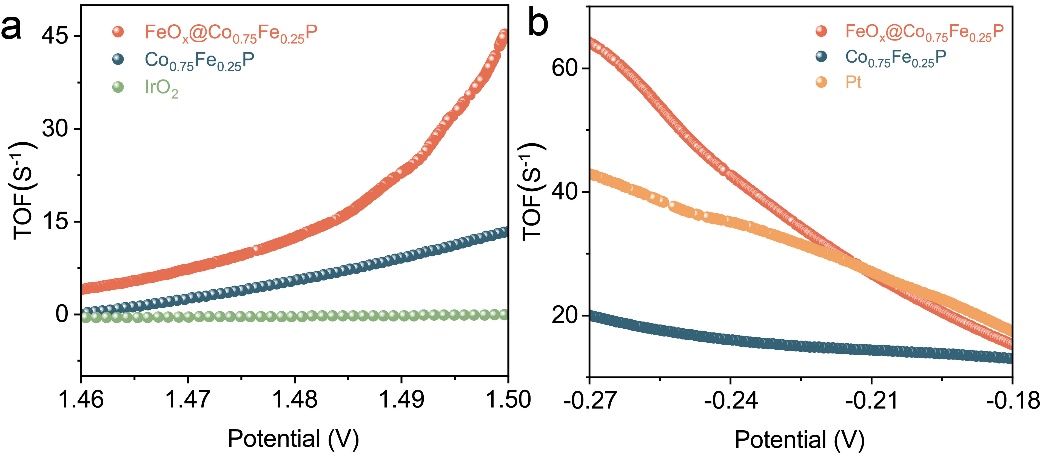


**Figure S9.** TOF values for (a) OER and (b) HER.


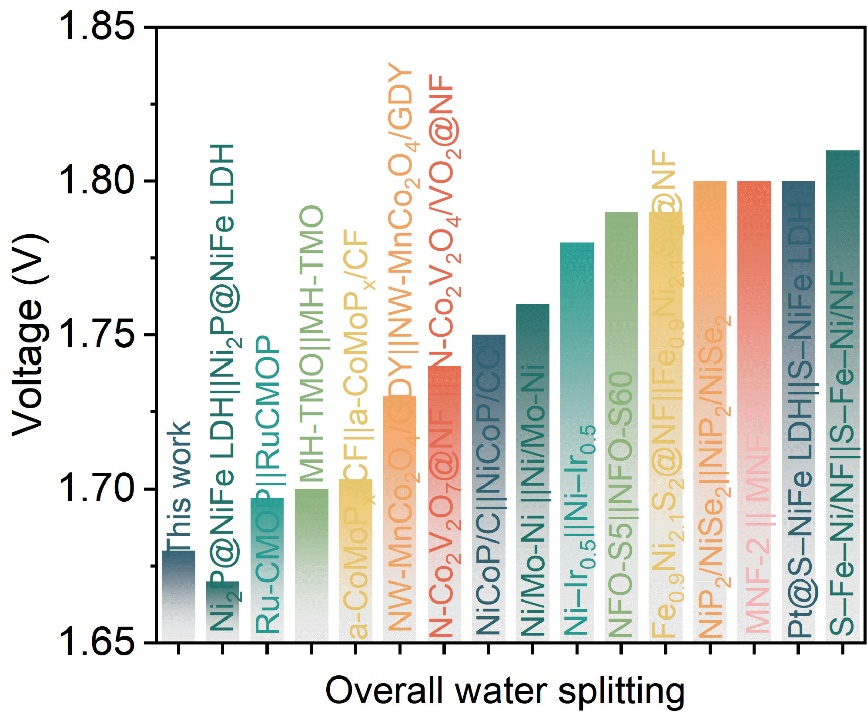


**Figure S10.** Comparison of the cell voltages at 100 mA cm^-2^ for FeO_x_@Co_0.75_Fe_0.25_P with reported electrocatalysts.





**Figure S11.** Catalytic performance comparison.


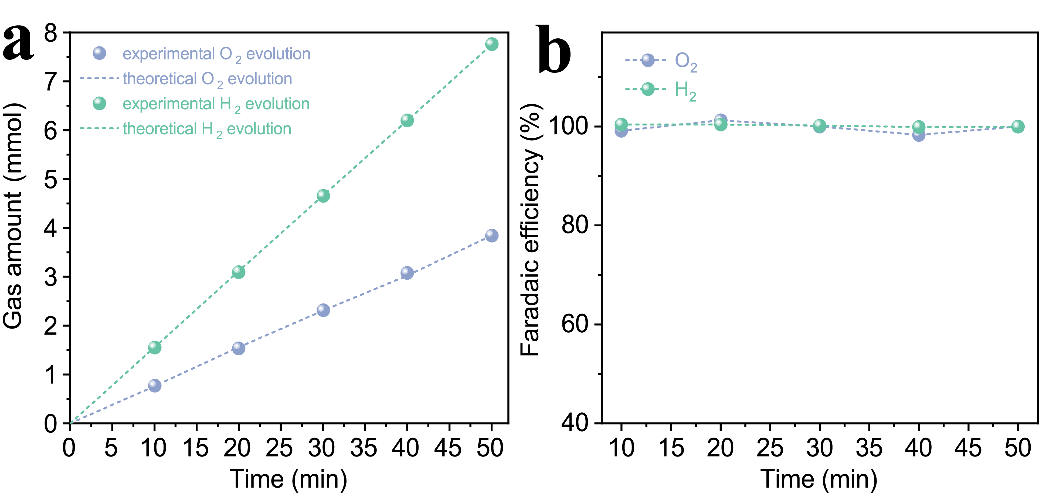


**Figure S12.** (a) Experimental and theoretical gas evolution of FeO_x_@Co_0.75_Fe_0.25_P‖FeO_x_@Co_0.75_Fe_0.25_P electrode pairs versus time. (b) Faradaic efficiency of H_2_ and O_2_.


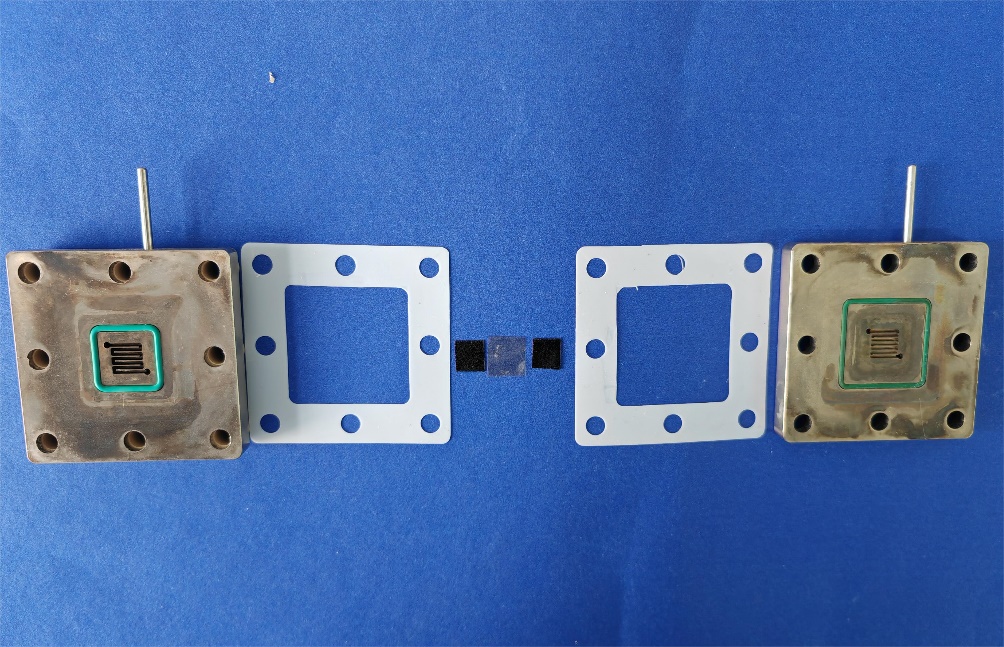


**Figure S13.** Optical photograph of the membrane electrode assembly.


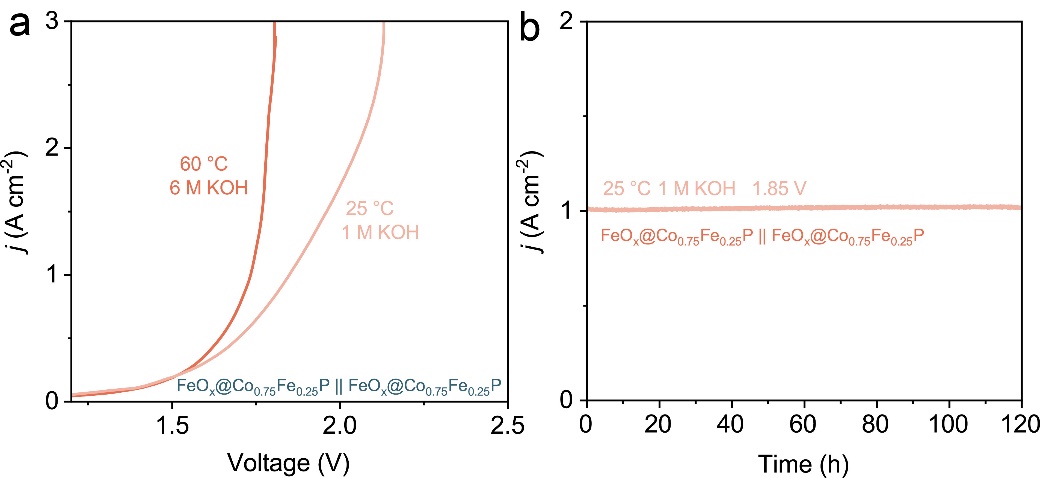


**Figure S14.** (a) LSV curves of the alkaline electrolyzer. (b) Long-term stability test.


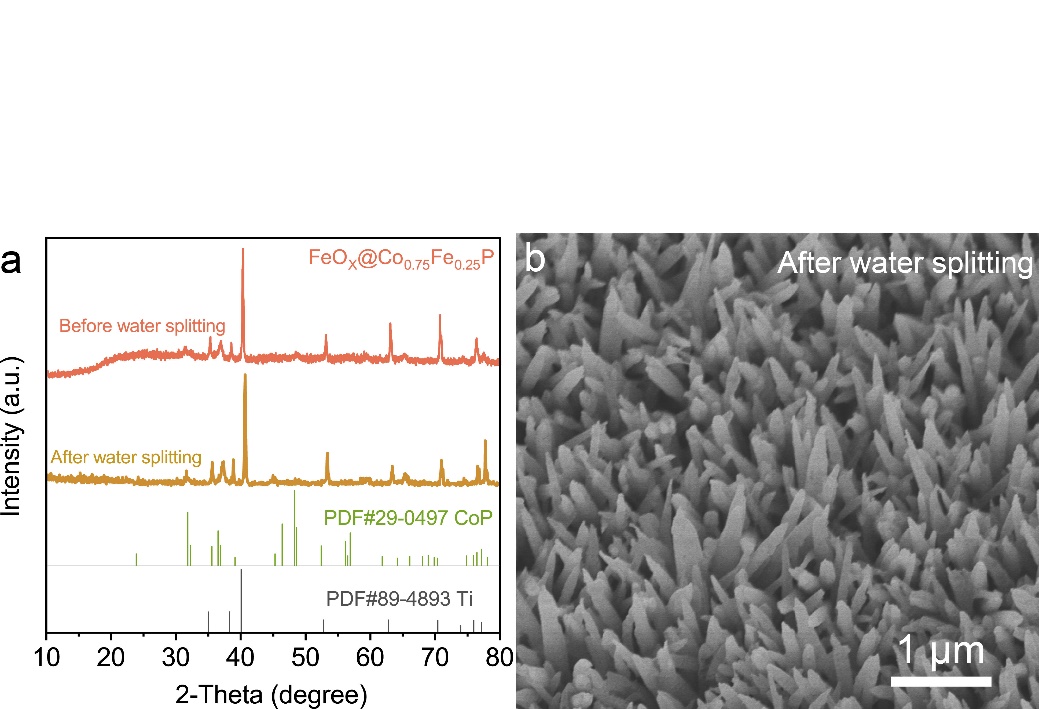


**Figure S15.** (a) XRD patterns and (b) SEM images of FeO_x_@Co_0.75_Fe_0.25_P after 120 h water splitting test. After a 120-hour water splitting test, there were no significant changes in the phase and morphology of the catalyst, indicating its robustness.


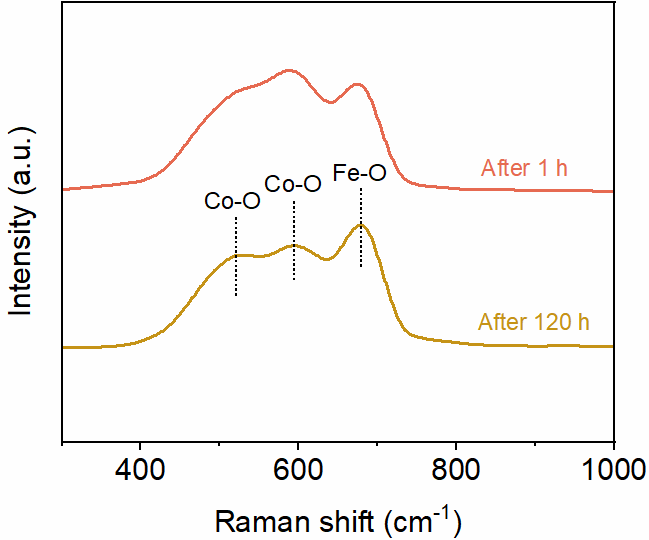


**Figure S16.** Raman spectra of pristine and recycled FeO_x_@Co_0.75_Fe_0.25_P.


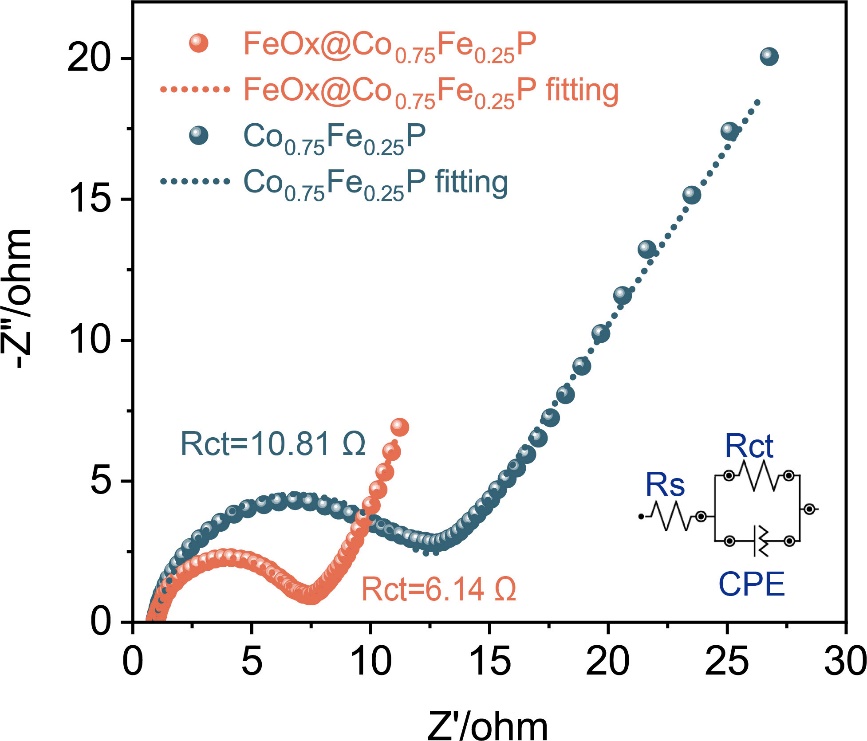


**Figure S17** Nyquist plot measured with an open-circuit voltage of 1.20 V and corresponding equivalent circuit. FeO_x_@Co_0.75_Fe_0.25_P exhibits reduced resistance, indicative of accelerated charge transfer. Additionally, its steeper slope in the low-frequency region of EIS suggests enhanced mass transfer in FeO_x_@Co_0.75_Fe_0.25_P.


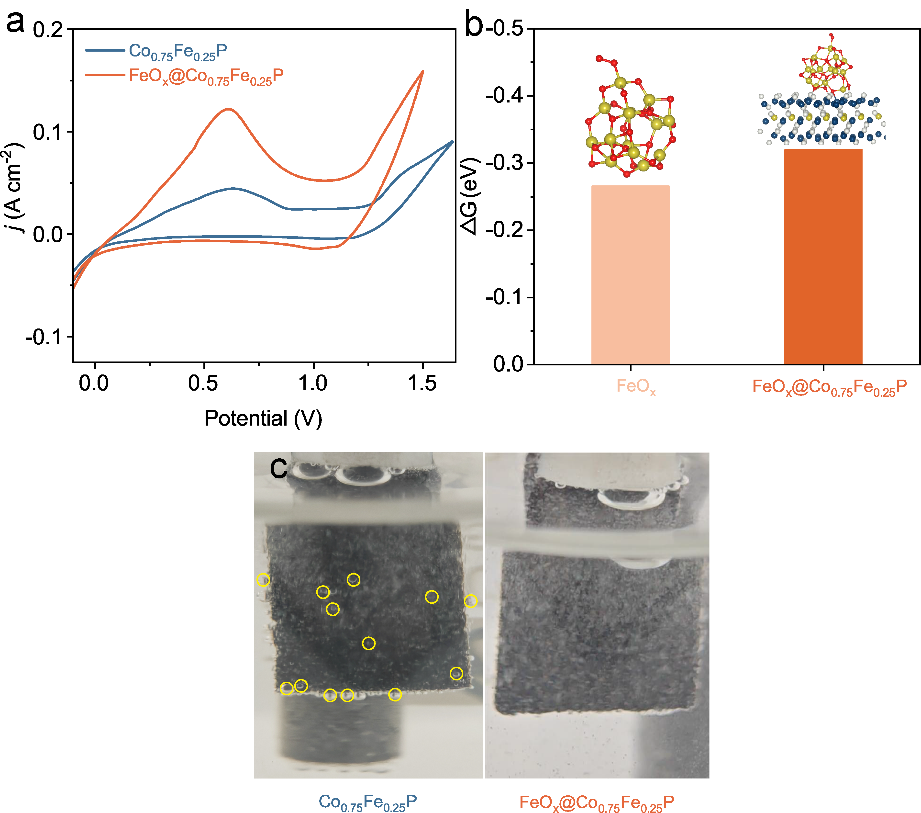


**Figure S18**. a) Cyclic voltammograms of FeO_x_@Co_0.75_Fe_0.25_P and Co_0.75_Fe_0.25_P. b) Theoretical calculation of the desorption energy of O_2_ from FeO_x_ and FeO_x_@Co_0.75_Fe_0.25_P. c) Optical photograph of the electrode surface at 100 mA cm^-2^.


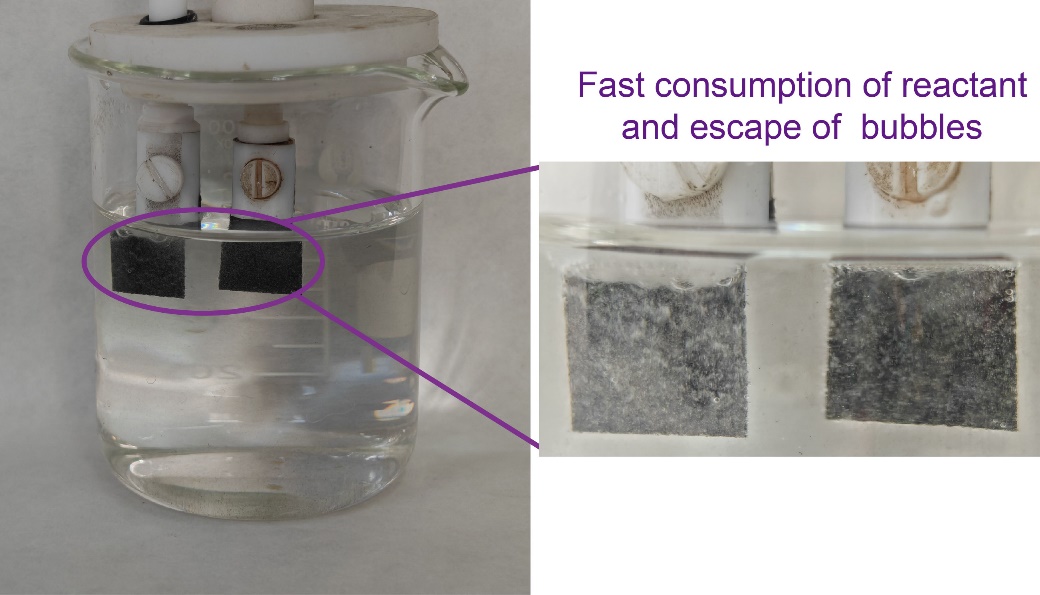


**Figure S19.** Optical photograph of a two-electrode electrolytic cell. At high current densities, bubbles fastly detach from the catalyst's surface, signifying a rapid mass transfer process occurring on the catalyst.


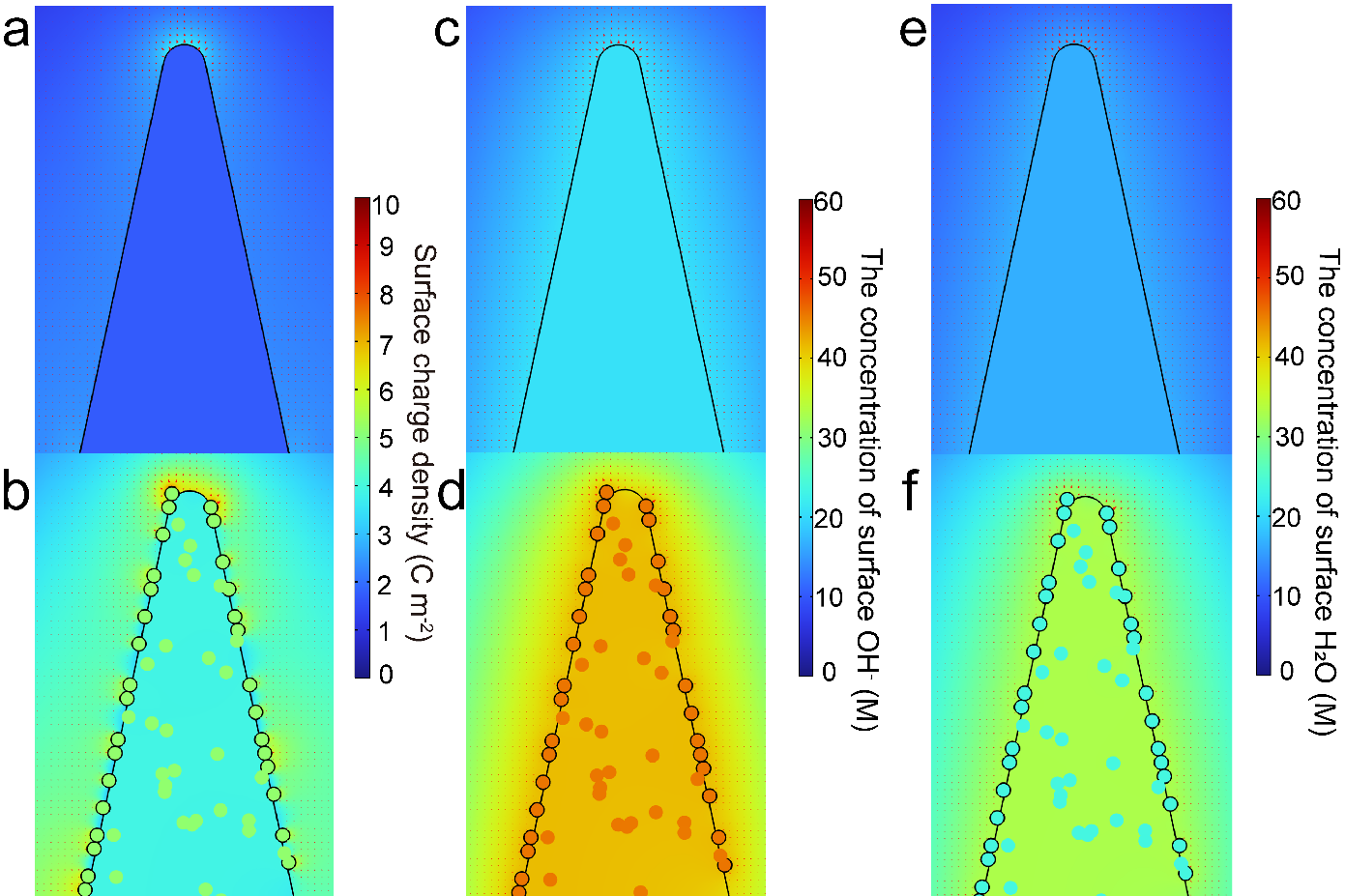


**Figure S20**. Charge density distribution on the surface of a) Co_0.75_Fe_0.25_OOH and b) FeO_x_@Co_0.75_Fe_0.25_OOH. Surface density distribution of OH^-^ on the electrode surface of c) Co_0.75_Fe_0.25_P and d) FeO_x_@Co_0.75_Fe_0.25_OOH. Surface H_2_O density distribution on the electrode surface of e) Co_0.75_Fe_0.25_OOH and f) FeO_x_@Co_0.75_Fe_0.25_OOH.


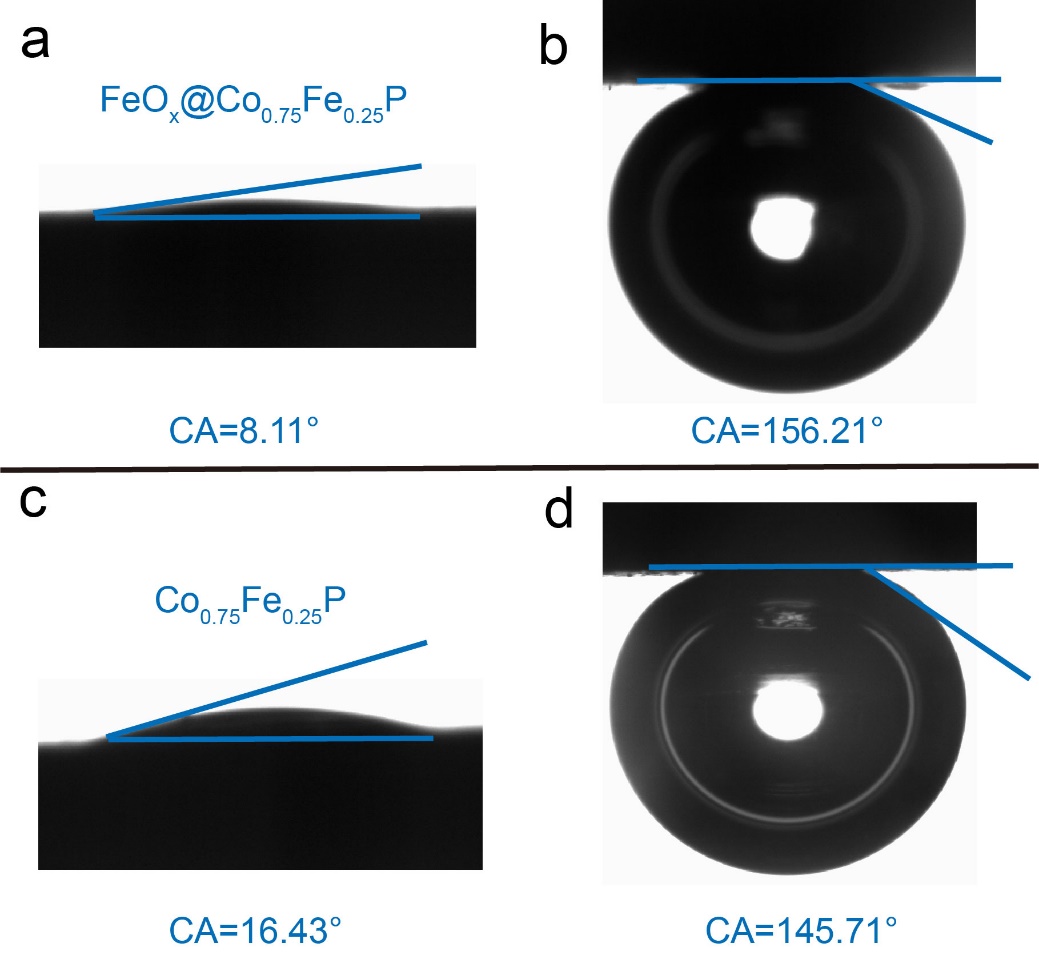


**Figure S21.** Water contact angle(CA) images of (a) FeO_x_@Co_0.75_Fe_0.25_P and (b) Co_0.75_Fe_0.25_P catalyst under water. Bubble contact angle images of (c) FeO_x_@Co_0.75_Fe_0.25_P and (d) Co_0.75_Fe_0.25_P catalyst under water. Compared to Co_0.75_Fe_0.25_P, FeO_x_@Co_0.75_Fe_0.25_P exhibits a reduced water contact angle and an increased bubble contact angle, demonstrating its enhanced hydrophilicity and gas-repelling characteristics.


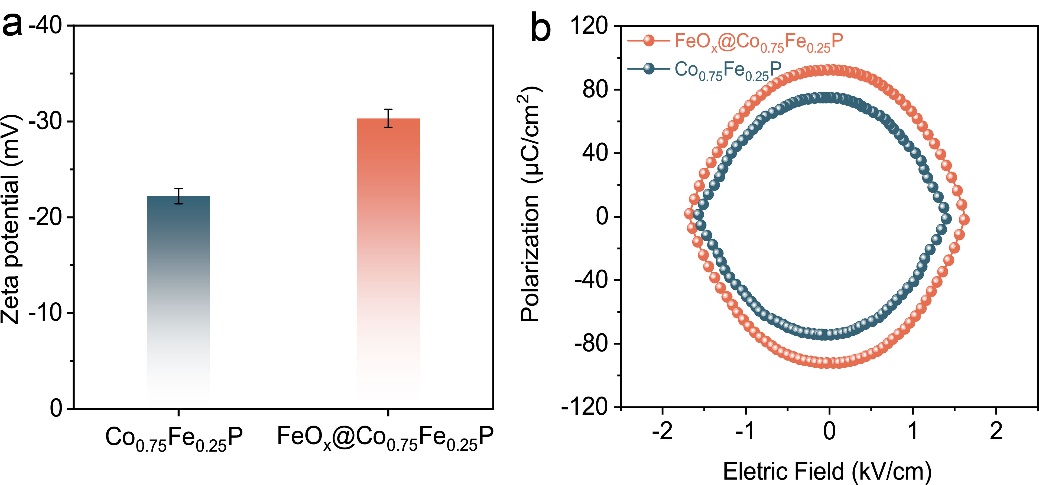


**Figure S22.** (a) Zeta potential and (b) Electric polarization curve of FeO_x_@Co_0.75_Fe_0.25_P.


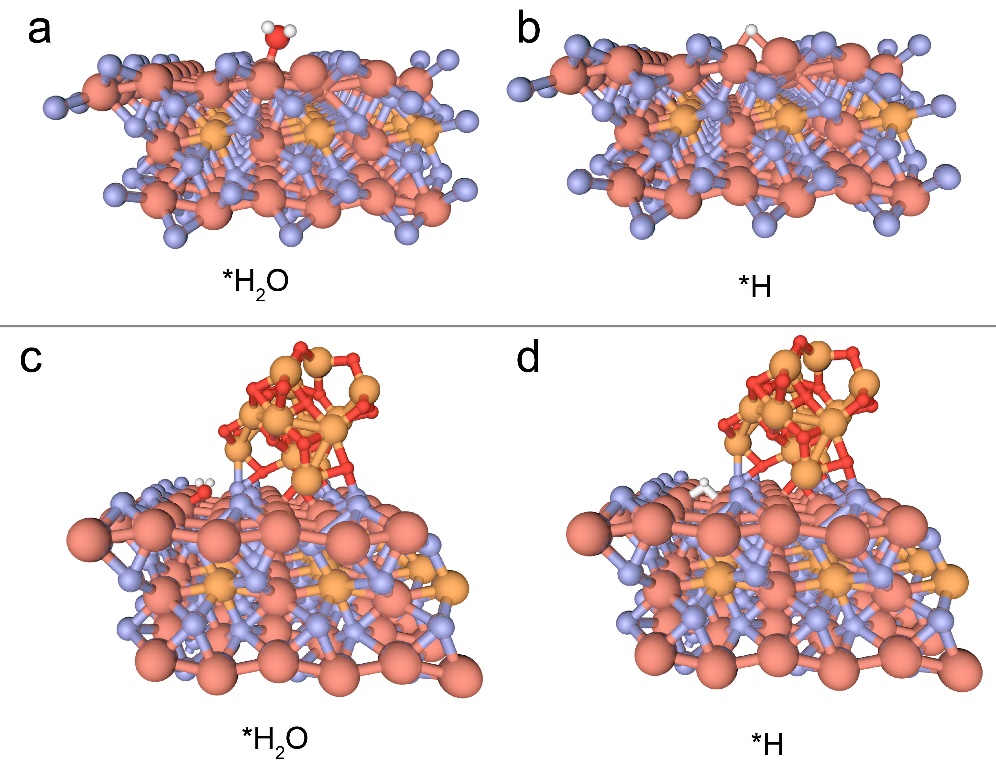


**Figure S23.** The optimized geometries for the crucial intermediate in the HER processes.


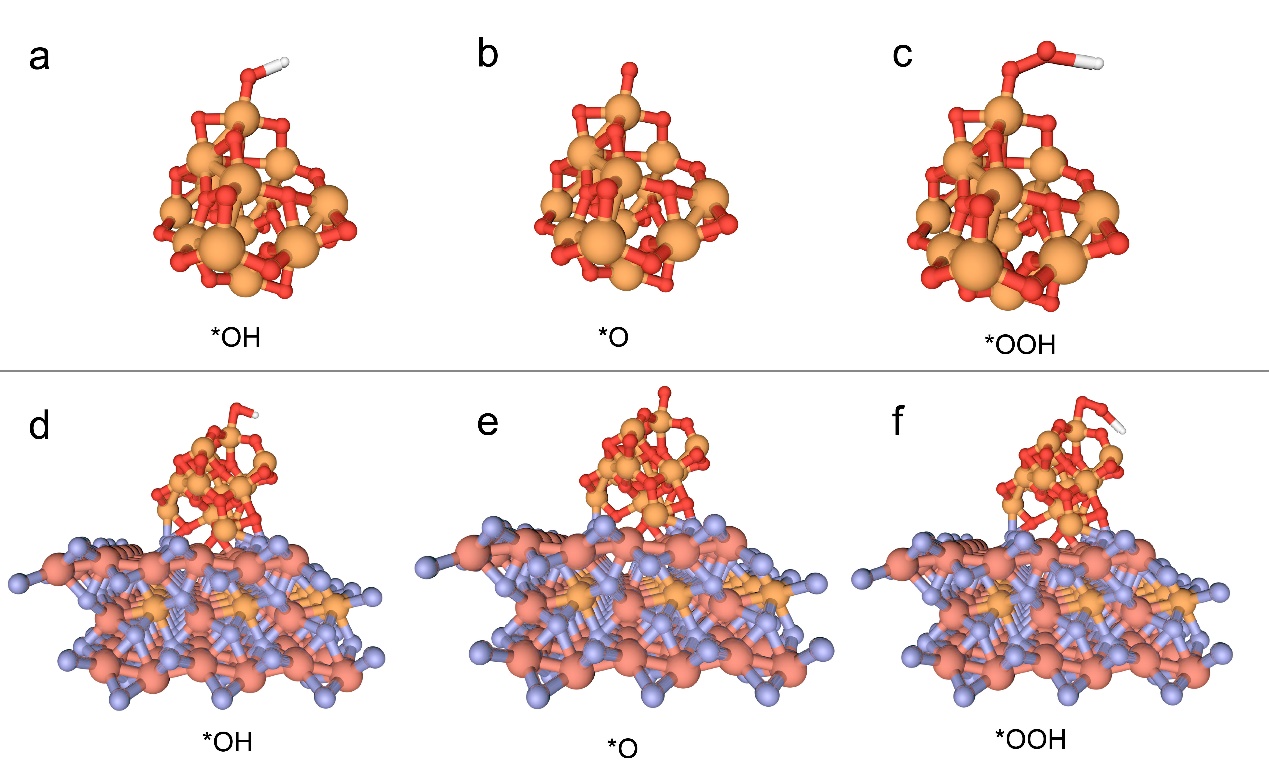


**Figure S24.** The optimized geometries for the crucial intermediate in the OER processes.


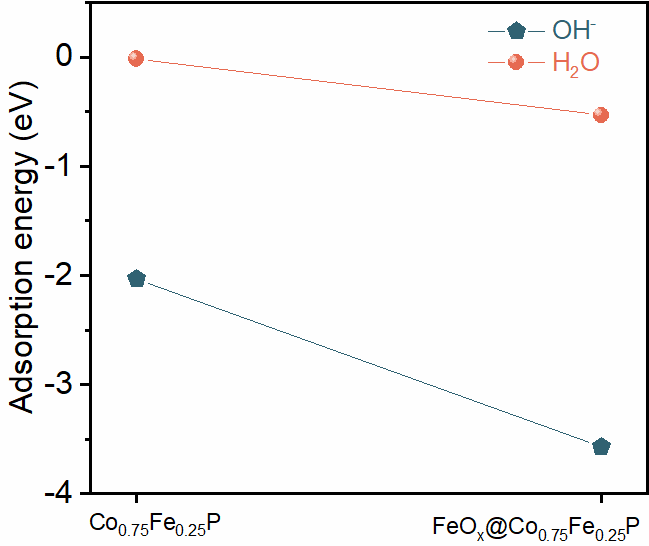


**Figure S25.** Adsorption energy of H_2_O and OH^-^.


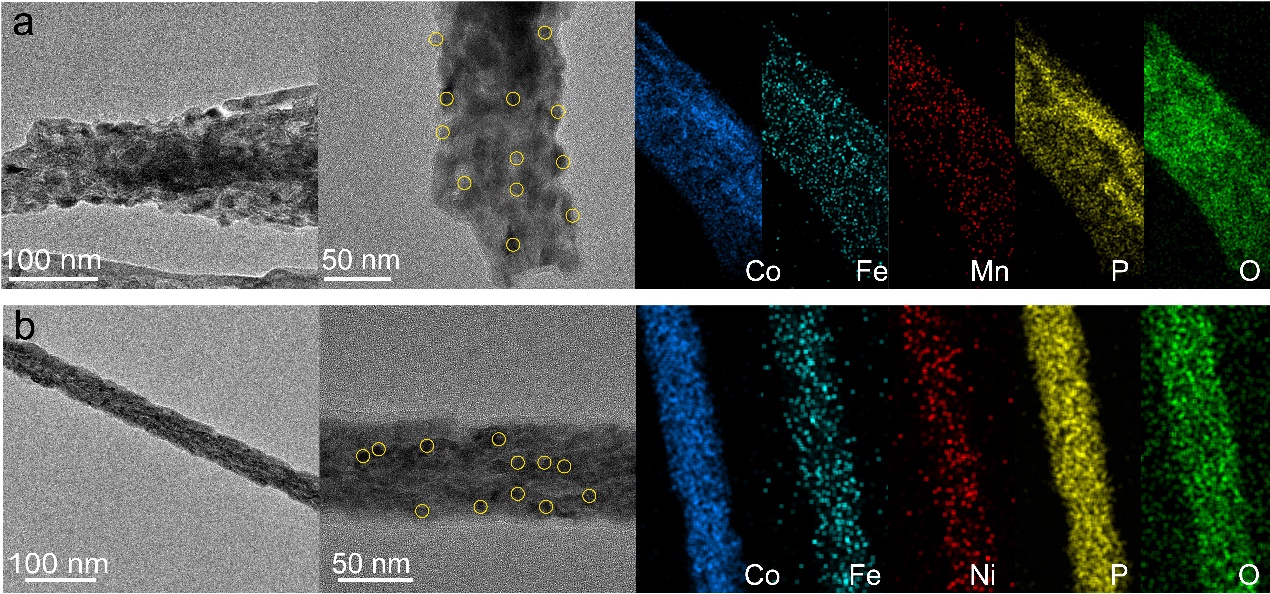


**Figure S26.** HRTEM image and element mapping of (a) MnO_x_@Co_0.75_Fe_0.25_P and (b) NiO_x_@Co_0.75_Fe_0.25_P.


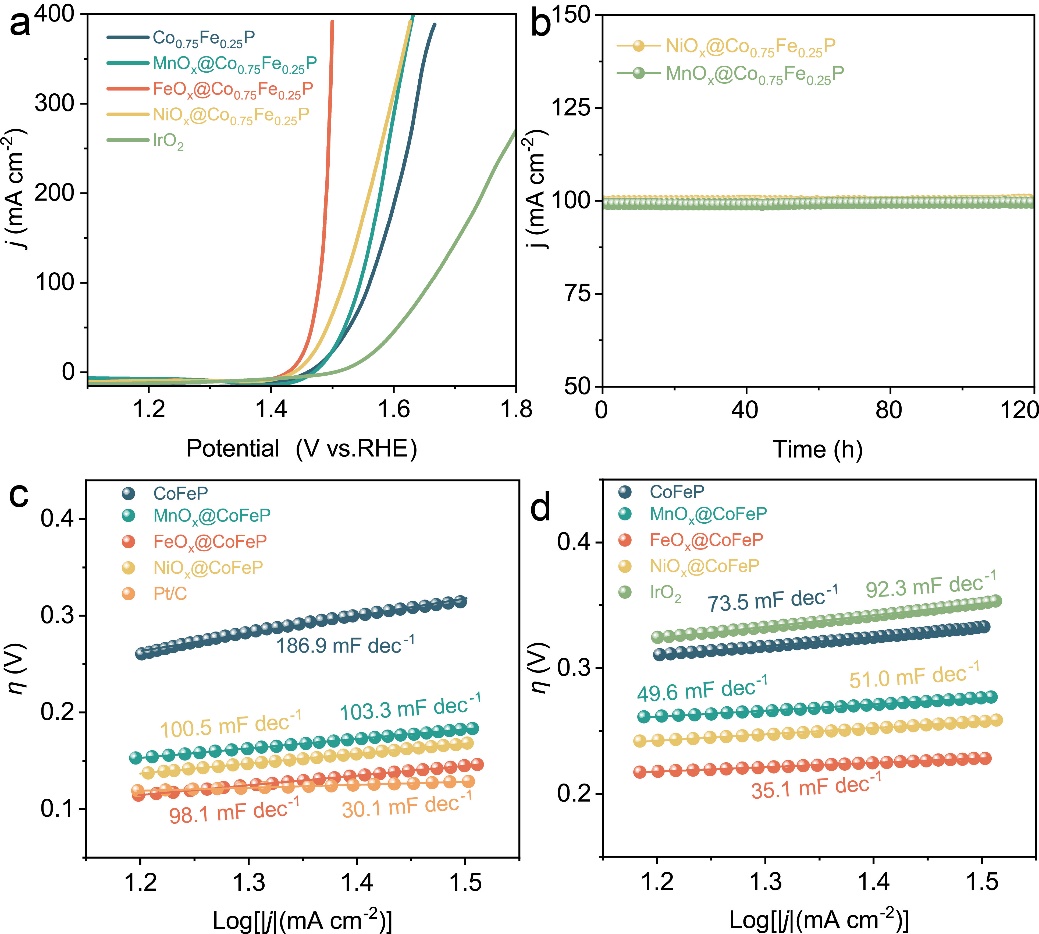


**Figure S27.** (a) LSV curve for catalyst. (b) i-t test. (c and d) The corresponding Tafel plots.


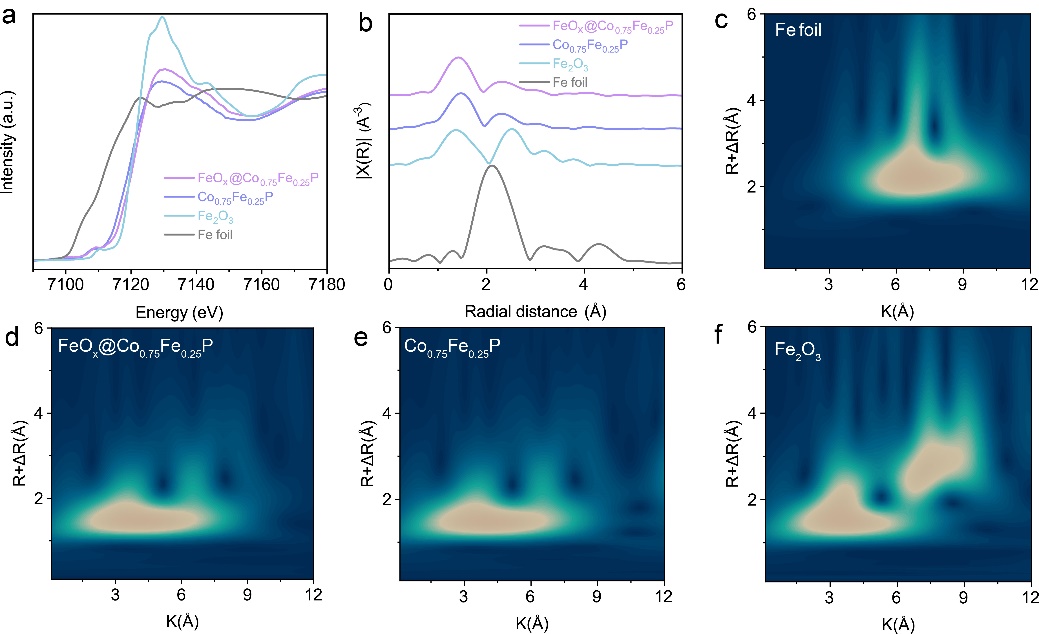


**Figure S28.** (a) Fe *K*-edge X-ray absorption near-edge structure spectra. (b) the corresponding FT- Fourier-transform extended X-ray absorption fine structure spectra. (c to f) WT-EXAFS plots.


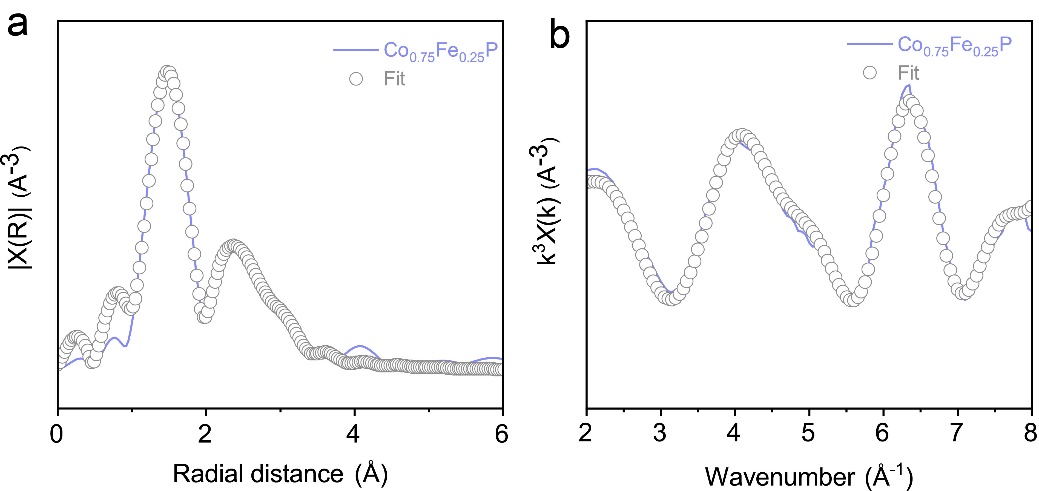


**Figure S29.** Fitting results of Co_0.75_Fe_0.25_P in (a) Fe *K* space and (b) *R* space.


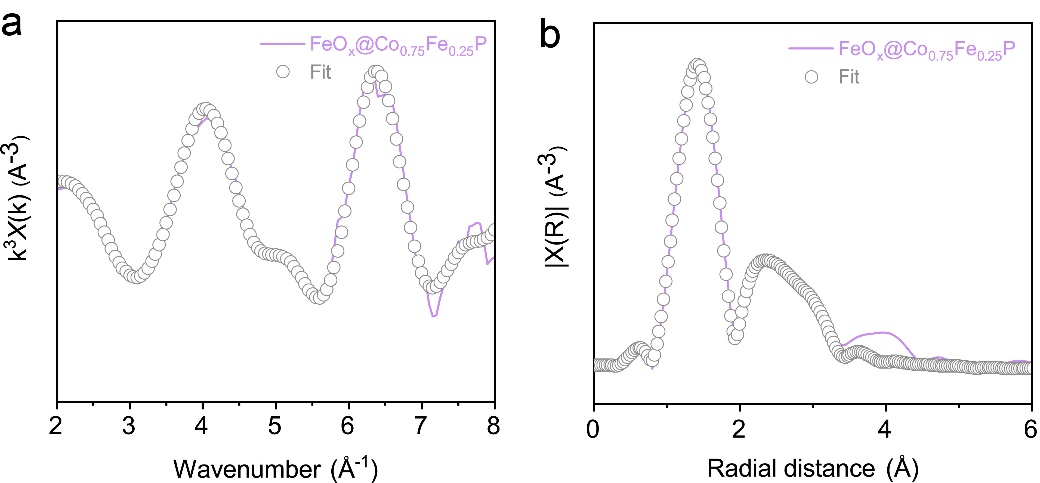


**Figure S30.** Fitting results of FeO_x_@Co_0.75_Fe_0.25_P in (a) Fe *K* space and (b) *R* space.


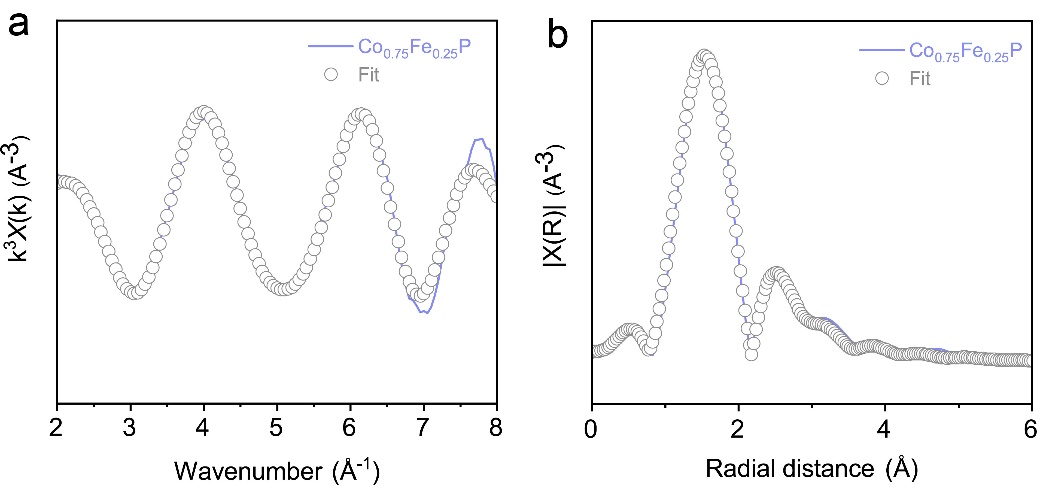


**Figure S31.** Fitting results of Co_0.75_Fe_0.25_P in (a) Co *K* space and (b) *R* space.


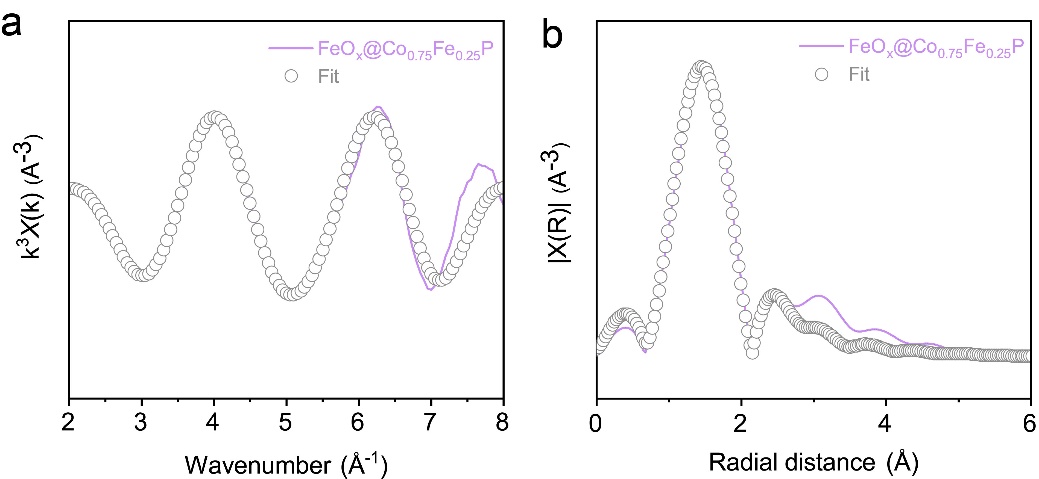


**Figure S32.** Fitting results of FeO_x_@Co_0.75_Fe_0.25_P in (a) Co *K* space and (b) *R* space.


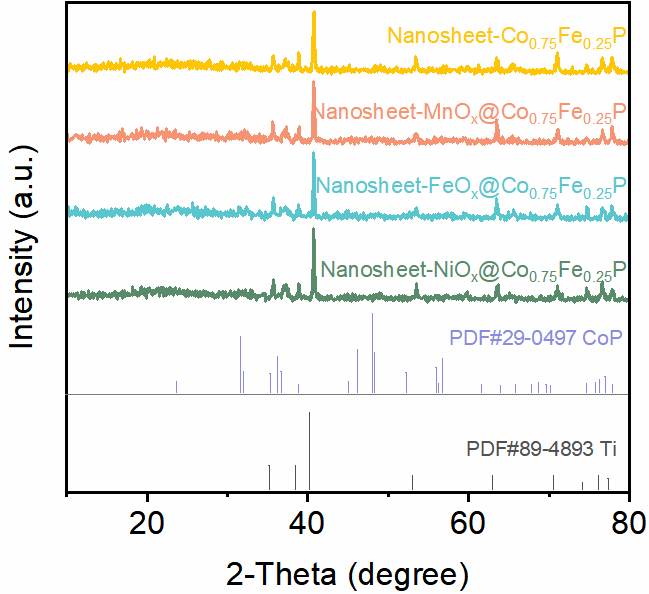


**Figure S33.** XRD for nanosheet catalyst. We have successfully synthesized Co_0.75_Fe_0.25_P nanosheets with various anchored clusters, each consistently exhibiting a phase that aligns with the standard card of CoP (PDF#29-0497).


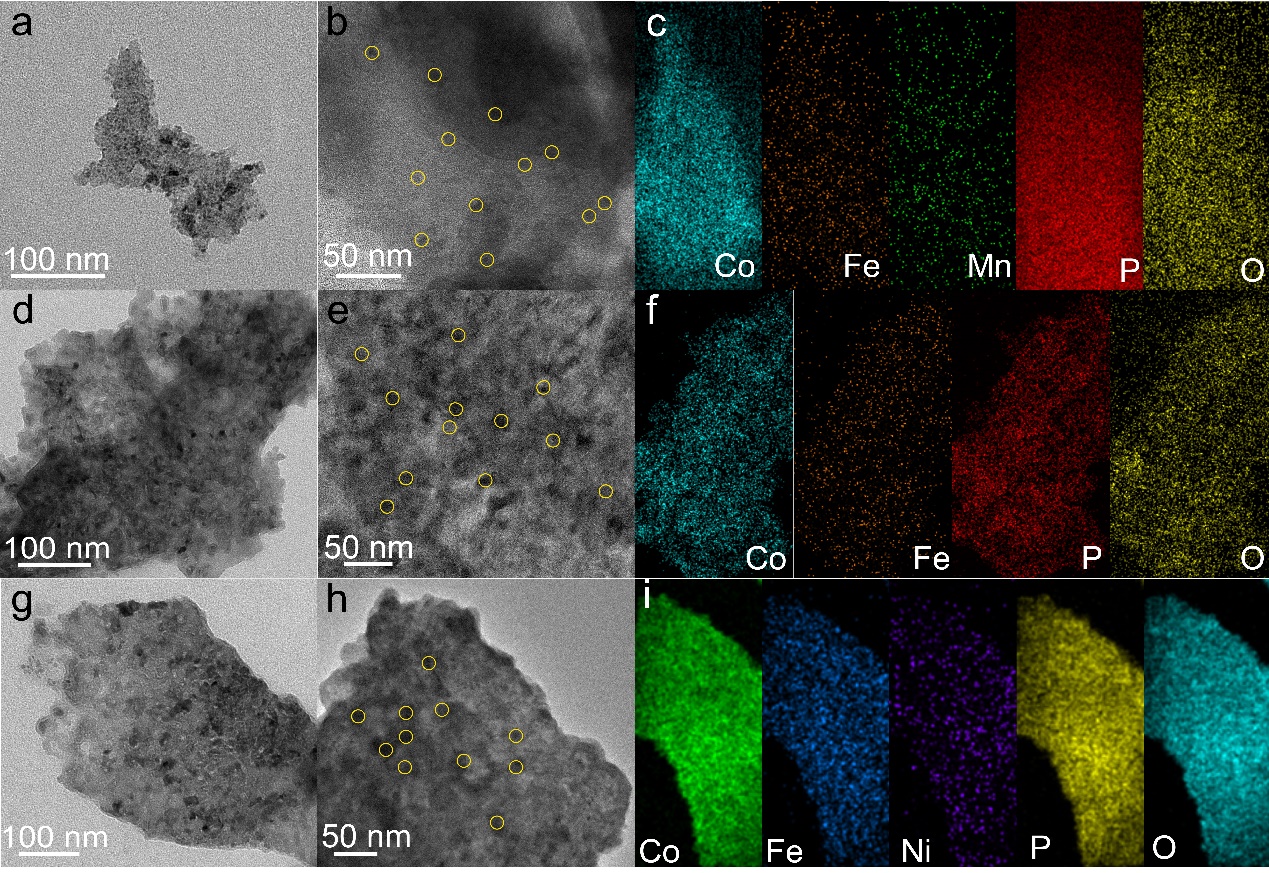


**Figure S34.** HRTEM image and element mapping for (a to c) MnO_x_@Co_0.75_Fe_0.25_P, (d to f) FeO_x_@Co_0.75_Fe_0.25_P and (g to i) NiO_x_@Co_0.75_Fe_0.25_P**.**


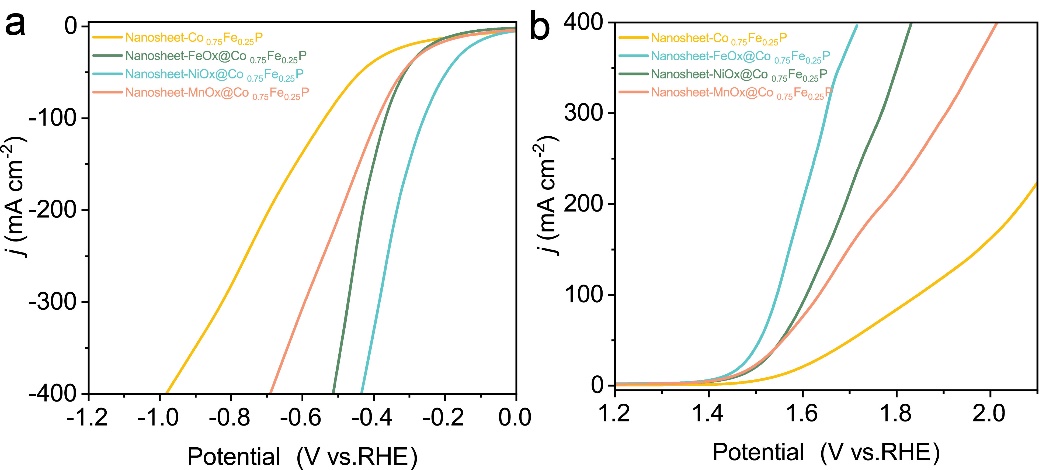


**Figure S35.** (a) HER and (b) OER curve for nanosheet catalysts. Similar in properties to the cluster-anchored Co_0.75_Fe_0.25_P nanorods, the cluster-anchored Co_0.75_Fe_0.25_P nanosheet exhibit superior qualities compared to Co_0.75_Fe_0.25_P. Among these, the FeO_x_@Co_0.75_Fe_0.25_P nanosheets display the most exceptional properties.


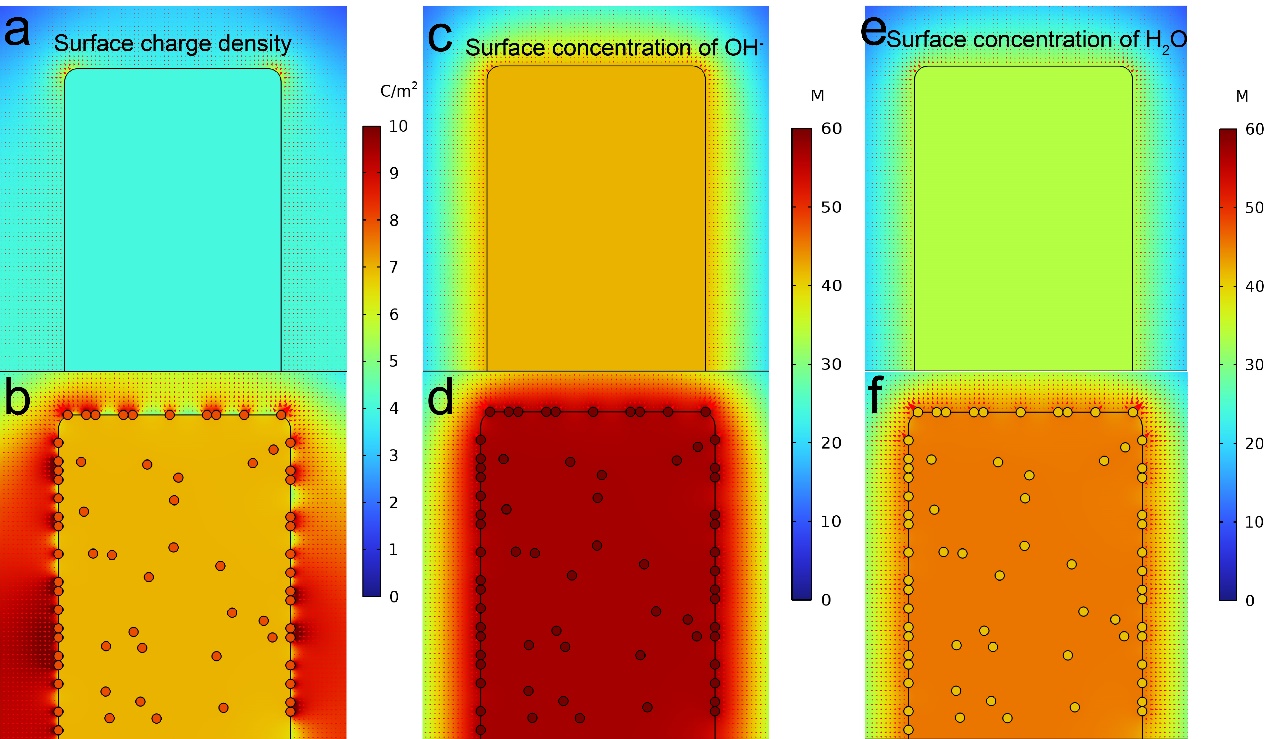


**Figure S36.** Charge density distribution on the surface of (a) Co_0.75_Fe_0.25_P and (b) FeO_x_@Co_0.75_Fe_0.25_P nanosheet. Surface OH^−^density distribution on the electrode surface of (c) Co_0.75_Fe_0.25_P and (d) FeO_x_@Co_0.75_Fe_0.25_P nanosheet. Surface H_2_O density distribution on the electrode surface of (e) Co_0.75_Fe_0.25_P and (f) FeO_x_@Co_0.75_Fe_0.25_P nanosheet.

**Table S1.** Calculation results from COMSOL.

|  | Surface charge density (C m^-2^ ) | Surface OH^-^ concentration (mol L^-1^) | Surface H_2_O concentration(mol L^-1^) |
| --- | --- | --- | --- |
| FeO_x_@Co_0.75_Fe_0.25_P | 8.25 | 59.96 | 43.65 |
| CoP | 1.15 | 8.55 | 10.61 |
| Co_0.875_Fe_0.125_P | 3.22 | 18.41 | 13.17 |
| Co_0.75_Fe_0.25_P | 4.26 | 24.21 | 22.84 |

**Table S2.** The EXAFS fitting parameters at the Fe *K*-edge for samples.

| Sample | Shell | *N* ^a^ | *R* (Å) ^b^ | ***σ***^2^ (Å2·10^-3^) ^c^ | Δ*E_0_* (eV) ^d^ | *R* factor |
| --- | --- | --- | --- | --- | --- | --- |
| Co_0.75_Fe_0.25_P | Fe-P | 4.2* | 2.40±0.24 | 3.70 | -8.0 | 0.0012 |
|  | Fe-Fe | 4.0* | 2.89±0.23 | 8.48 | -8.0 |  |
| FeO_x_@Co_0.75_Fe_0.25_P | Fe-P | 4.0* | 2.31±0.22 | 2.48 | 2.83 | 0.0096 |
|  | Fe-O | 3.8* | 1.96±0.01 | 0.525 | 2.83 |  |
|  | Fe-Fe | 4.0* | 3.03±0.36 | 1.55 | 2.83 |  |

*Note: ^a^* *N*: coordination numbers; *^b^* *R*: bond distance; *^c^* *σ*^2^: Debye-Waller factors; *^d^* Δ*E*_0_: the inner potential correction. *R* factor: goodness of fit.

**Table S3.** The EXAFS fitting parameters at the Co *K*-edge for samples.

| Sample | Shell | *N* ^a^ | *R* (Å) ^b^ | ***σ***^2^ (Å^2^·10^-3^) ^c^ | Δ*E_0_* (eV) ^d^ | *R* factor |
| --- | --- | --- | --- | --- | --- | --- |
| Co_0.75_Fe_0.25_P | Co-P | 3.9* | 2.34±0.06 | 3.70 | -8.0 | 0.0012 |
|  | Co-Co | 3* | 2.94±0.15 | 8.48 | -8.0 |  |
| FeO_x_@Co_0.75_Fe_0.25_P | Co-P | 3.9* | 2.37±0.09 | 3.72 | -8.8 | 0.0012 |
|  | Co-Co | 3* | 2.90±0.11 | 8.56 | -8.8 |  |

*Note: ^a^* *N*: coordination numbers; *^b^* *R*: bond distance; *^c^* *σ*^2^: Debye-Waller factors; *^d^* Δ*E*_0_: the inner potential correction. *R* factor: goodness of fit.

**Table S4.** Elemental analysis of nanorod by ICP-MS.

|  | Co(wt%) | Fe(wt%) | Mn/Ni(wt%) |
| --- | --- | --- | --- |
| CoP | 71.410 | - | - |
| Co_0.875_Fe_0.125_P | 61.524 | 9.025 | - |
| Co_0.75_Fe_0.25_P | 54.025 | 17.258 | - |
| Co_0.625_Fe_0.375_P | 44.025 | 25.052 | - |
| Co_0.5_Fe_0.5_P | 37.025 | 34.982 | - |
| FeO_x_@Co_0.75_Fe_0.25_P | 54.112 | 21.526 | - |
| MnO_x_@Co_0.75_Fe_0.25_P | 53.981 | 17.335 | 4.255 |
| NiO_x_@Co_0.75_Fe_0.25_P | 54.041 | 17.786 | 4.317 |

**Table S5.** Elemental analysis of nanosheet by ICP-MS.

|  | Co(wt%) | Fe(wt%) | Mn/Ni(wt%) |
| --- | --- | --- | --- |
| Co_0.75_Fe_0.25_P | 55.011 | 17.998 | - |
| FeO_x_@Co_0.75_Fe_0.25_P | 54.856 | 22.516 | - |
| MnO_x_@Co_0.75_Fe_0.25_P | 54.982 | 17.152 | 4.685 |
| NiO_x_@Co_0.75_Fe_0.25_P | 54.995 | 17.685 | 4.415 |

**Table S6.** Ground state energies of basic species calculated by DFT.

| Species | formula unit (f.u.) | *E*_total_(eV) | E_f.u._(eV) |
| --- | --- | --- | --- |
| Co | 2 | -14.071 | -7.036 |
| Fe | 2 | -16.476 | -8.238 |
| P | 8 | -42.985 | -5.373 |
| CoP | 4 | -54.427 | -13.607 |
| FeP | 4 | -59.400 | -14.850 |
| Co_0.75_Fe_0.25_P | 4 | -89.349 | -22.337 |

**Table S7.** Total energies of clean Co_0.75_Fe_0.25_P and FeO_x_@Co_0.75_Fe_0.25_P, as well as energies of the most stable absorption geometries for H_2_O* and H* intermediates.

| Surfaces | *E*(H_2_O*)/eV | *E*(H*)/eV |
| --- | --- | --- |
| Co_0.75_Fe_0.25_P | -1274.355 | -1283.541 |
| FeO_x_@Co_0.75_Fe_0.25_P | -1627.642 | -1615.166 |

**Table S8.** Total energies of clean FeO_x_ and FeO_x_@Co_0.75_Fe_0.25_P, as well as energies of the most stable absorption geometries for O*, OH*, OOH*, and OO* intermediates.

| Surfaces | *E*(*)/eV | *E*(O*)/eV | *E*(OH*)/eV | *E*(OOH*)/eV | *E*(OO*)/eV |
| --- | --- | --- | --- | --- | --- |
| FeO_x_ | -264.272 | -269.550 | -274.467 | -278.731 | -274.530 |
| FeO_x_@Co_0.75_Fe_0.25_P | -1612.001 | -1617.430 | -1622.209 | -1626.542 | -1622.315 |

**Table S9.** The ZPE and entropy corrections for H_2_O* and H* intermediates on Co_0.75_Fe_0.25_P and FeO_x_@Co_0.75_Fe_0.25_P.

| Surfaces | *ZPE*(H_2_O*)/eV | *ZPE*(H*)/eV | *TS*(H_2_O*)/eV | *TS*(H*)/eV |
| --- | --- | --- | --- | --- |
| Co_0.75_Fe_0.25_P | 0.745 | 0.188 | 0.11 | 0.009 |
| FeO_x_@Co_0.75_Fe_0.25_P | 0.768 | 0.196 | 0.113 | 0.011 |

**Table S10.** The ZPE and entropy corrections for O*, OH*, and OOH* intermediates on FeO_x_ and FeO_x_@Co_0.75_Fe_0.25_P.

| Surfaces | *ZPE*(O*)/eV | *ZPE*(OH*)/eV | *ZPE*(OOH*)/eV | *TS*(O*)/eV |
| --- | --- | --- | --- | --- |
| FeO_x_ | 0.106 | 0.392 | 0.500 | 0.089 |
| FeO_x_@Co_0.75_Fe_0.25_P | 0.235 | 0.429 | 0.537 | 0.050 |
|  | *TS*(OH*)/eV | *TS*(OOH*)/eV | *ZPE*(OO*)/eV | *TS*(OO*)/eV |
| FeO_x_ | 0.039 | 0.095 | 0.222 | 0.160 |
| FeO_x_@Co_0.75_Fe_0.25_P | 0.092 | 0.158 | 0.208 | 0.144 |

**Table S11.** Comparison of OER/HER for FeO_x_@Co_0.75_Fe_0.25_P with analogues

| Electrocatalyst | OER *η*_100_(mV) | HER *η*_100_(mV) | References |
| --- | --- | --- | --- |
| Ni_2_P-Fe_2_P/NF | 230 | 235 | Adv. Funct. Mater. 2021, 31, 2006484 |
| Cu_3_P/Ni_2_P@CF | 400 | 210 | Chem. Eng. J. 2022, 448, 137706 |
| Fe–Ni_2_P@PC/Cu_x_S | 390 | 310 | Nano Energy 2021, 84, 105861 |
| Fe-Ni_2_P/Ni_5_P_4_@N-C | 340 | 330 | Inorg. Chem. 2023, 62, 6518-6526 |
| NiCoP-120 | 321 | 150 | Adv. Energy Mater. 2023, 13, 2300499 |
| Ru-MoCoP | 340 | 180 | Adv. Funct. Mater. 2024, 34, 2309330 |
| Ni_2_P@C-350 | 430 | 220 | Adv. Mater. Interfaces 2022, 9, 2200673 |
| Ni-N,P/CNFs | 390 | 350 | Nano Energy 2022, 98, 107266 |
| Ni_2_P-CoCH/CFP | 320 | 143 | Angew. Chem. 2023, 135, e202302795 |
| FeCo-P | 270 | 290 | Adv. Sci. 2024, 11, 2306919 |
| NiFe-MS/MOF@NF | 243 | 205 | Adv. Sci. 2020, 7, 2001965 |
| Co_9_S_8_-Fe@CNT | 230 | 298 | Adv. Mater. 2024, 36, 2306138 |
| Ir@Zr-CoP | 370 | 175 | Adv. Energy Mater. 2023, 13, 2301841 |
| PtP_2_/CoP | 250 | 101 | Adv. Funct. Mater. 2024, 2313935 |
|  |  |  |  |
| N-CoP/CeO_2_ | 280 | 217 | Chem. Eng. J. 2023, 460, 141119 |
| CoTe_2_/CoP | 354 | 148 | Appl. Catal. B Environ. 2023, 329, 122551 |
| Co_2_N/CoP@CC | 320 | 105 | Chem. Eng. J. 2023, 470, 144242 |
| DH-CuCo-P@NC/CC | 339 | 176 | Appl. Catal. B Environ. 2023, 325, 122295 |
| FeO_x_@Co_0.75_Fe_0.25_P | 240 | 200 | This work |

**Table S12.** Comparison of water splitting performances for FeO_x_@Co_0.75_Fe_0.25_P|| FeO_x_@Co_0.75_Fe_0.25_P with the benchmarking catalysts

| Electrocatalyst | Potential  at 100 mA cm^-2^ (V) | Reference |
| --- | --- | --- |
| FeCoNiMnRu/CNFs||FeCoNiMnRu/CNFs | 1.65 | Nat. Commun. **2022**,13, 2662 |
| MAPbBr_3_@AlPO-5||Pt | 1.63 | Adv. Mater. **2023**, 35, 2301166 |
| Ru-CMOP||RuCMOP | 1.697 | Nano Energy **2022,**101,107566 |
|  |  |  |
| a-CoMoP_x_/CF|| a-CoMoP_x_/CF | 1.703 | Adv. Funct. Mater. **2020**, 2003889 |
| NiCoP/C||NiCoP/CC | 1.75 | ACS Catal. **2017**, 7, 4131 |
| Ni/Mo-Ni ||Ni/Mo-Ni | 1.76 | Chem. Eng. J. **2022**,l435,134860 |
| Ni–Ir_0.5_||Ni–Ir_0.5_ | 1.78 | Inorg. Chem. Front. **2022**, 9, 6225 |
| NFO-S5||NFO-S60 | 1.79 | Appl. Catal. B **2022**,305, 121030 |
| Fe_0.9_Ni_2.1_S_2_@NF||Fe_0.9_Ni_2.1_S_2_@NF | 1.79 | Adv. Energy Mater. **2020**, 2001963 |
| MH-TMO||MH-TMO | 1.7 | Adv. Energy Mater. **2022**, 12, 2200067 |
| NiP_2_/NiSe_2_ || NiP_2_/NiSe_2_ | 1.8 | Appl. Catal. B. **2021**, 282, 119584 |
| N-Co_2_V_2_O_7_@NF || N-Co_2_V_2_O_4_/VO_2_@NF | 1.74 | J. Colloid Interf. Sci. **2023**, 629,111–120 |
| MNF-2 || MNF-2 | 1.8 | Appl. Catal. B. **2023**, 322, 122103 |
| Pt@S–NiFe LDH|| S–NiFe LDH | 1.8 | Adv. Mater. **2023**, 35, 2208209 |
| S–Fe–Ni/NF||S–Fe–Ni/NF | 1.81 | J. Mater. Chem. A, **2023**, 11, 4661 |
| Co-NC/CP|| Co-NC/CP | 1.86 | Energy Environ. Sci. **2020**,13, 545–553 |
| NW-MnCo_2_O_4_/GDY||NW-MnCo_2_O_4_/GDY | 1.73 | Adv. Funct. Mater. **2022**, 32, 2107179 |
| MoS_2_/NiFe LDH|| MoNiFe | 1.73 | Nat. Commun. **2022**,13, 2191 |
| FeO_x_@Co_0.75_Fe_0.25_P|| FeO_x_@Co_0.75_Fe_0.25_P | 1.67 | This work |

**References**

[1] B. Ravel, M. Newville, *J. Synchrotron Radiat.* **2005**, *12*, 537-541.

[2] Y. Hu, M. Liu, Q. Yang, L. Kong, L. Kang, *J. Energy Chem.* **2017**, *26*, 49-55.

[3] G. Kresse, J. Furthmüller, Comput. Mater. Sci **1996**, *6*, 15-50.

[4] G. Kresse, J. Hafner, Phys. Rev. B **1993**, *48*, 13115-13118.

[5] I. C. Man, H.-Y. Su, F. Calle-Vallejo, H. A. Hansen, J. I. Martínez, N. G. Inoglu, J. Kitchin, T. F. Jaramillo, J. K. Nørskov, J. Rossmeisl, *ChemCatChem* **2011**, *3*, 1159-1165.
